# Lymphotoxin, but Not TNF, Is Required for Prion Invasion of Lymph Nodes

**DOI:** 10.1371/journal.ppat.1002867

**Published:** 2012-08-09

**Authors:** Tracy O'Connor, Nathalie Frei, Jana Sponarova, Petra Schwarz, Mathias Heikenwalder, Adriano Aguzzi

**Affiliations:** Institute of Neuropathology, University Hospital of Zürich, Zürich, Switzerland; University of Edinburgh, United Kingdom

## Abstract

Neuroinvasion and subsequent destruction of the central nervous system by prions are typically preceded by a colonization phase in lymphoid organs. An important compartment harboring prions in lymphoid tissue is the follicular dendritic cell (FDC), which requires both tumor necrosis factor receptor 1 (TNFR1) and lymphotoxin β receptor (LTβR) signaling for maintenance. However, prions are still detected in *TNFR1^−/−^* lymph nodes despite the absence of mature FDCs. Here we show that TNFR1-independent prion accumulation in lymph nodes depends on LTβR signaling. Loss of LTβR signaling, but not of TNFR1, was concurrent with the dedifferentiation of high endothelial venules (HEVs) required for lymphocyte entry into lymph nodes. Using luminescent conjugated polymers for histochemical PrP^Sc^ detection, we identified PrP^Sc^ deposits associated with HEVs in *TNFR1^−/−^* lymph nodes. Hence, prions may enter lymph nodes by HEVs and accumulate or replicate in the absence of mature FDCs.

## Author Summary

Prions are unique infectious agents thought to be composed entirely of an abnormal conformer of the endogenous prion protein. Prions cause a severe neurological disorder in humans and other animals known as prion disease. Though prion disease can arise spontaneously or from genetic mutations in the gene encoding the prion protein, many cases of prion disease arise due to peripheral exposure to the infectious agent. In these cases, prions must journey from the gastrointestinal tract and/or the bloodstream to the brain. Prions often colonize secondary lymphoid organs prior to invading the nervous system *via* neighboring peripheral nerves. Prion accumulation in follicular dendritic cells found in germinal centers of lymphoid organs is thought to be a crucial step in this process. However, prion colonization of lymph nodes, in contrast to spleen, does not depend on follicular dendritic cells, indicating that other mechanisms must exist. Here, we identify the signaling pathway required for follicular dendritic cell-independent prion colonization of lymph nodes, which also controls the differentiation of high endothelial venules, the primary entry point for lymphocytes into lymph nodes. Importantly, prions could be found within these structures, indicating that high endothelial venules are required for prion entry and accumulation in lymph nodes.

## Introduction

Prions are unusual infectious agents thought to be comprised solely of an abnormally folded, aggregated isoform (PrP^Sc^) of the endogenous cellular prion protein (PrP^C^) [1]. Deposition of PrP^Sc^ aggregates, vacuolation, and neuronal loss in brain tissue are histopathological hallmarks of a group of neurological disorders collectively known as prion diseases or transmissible spongiform encephalopathies (TSEs), including scrapie in sheep, bovine spongiform encephalopathy (BSE) in bovids, chronic wasting disease (CWD) in cervids, and Creutzfeldt-Jakob disease (CJD) in humans [2].

Although TSEs seem to selectively damage the central nervous system (CNS), peripheral prion exposure leads to the accumulation of prions and PrP^Sc^ in secondary lymphoid organs (SLOs) long before neurological symptoms appear [3], [4], [5], [6], [7], [8], and it is largely from these extraneural sites that prions transmigrate to the peripheral nervous system (PNS) and finally the CNS [9], [10], [11]. Extraneural prion accumulation is thought to occur primarily within stromal cells found in germinal centers of lymphoid follicles known as follicular dendritic cells (FDCs) [4], [12], [13], [14], [15], [16], [17]. Maintenance of mature FDC networks depends on signaling through FDC-expressed lymphotoxin β receptor (LTβR) and tumor necrosis factor receptor 1 (TNFR1) by B cell-derived tumor necrosis factor (TNF) and lymphotoxins (LT) α and β [18], [19], [20], [21], [22], [23]. Accordingly, ablation of B cells, and hence loss of LTα/β and TNFα ligands, antagonizes prion deposition in secondary lymphoid organs [24], [25], and intraperitoneal (i.p.) injection of mice with TNFR1 or LTβR blocking antibodies prior to peripheral prion inoculation causes transient de-differentiation of FDCs, reduces splenic prion accumulation, and delays prion neuroinvasion [26], [27], [28], [29].

However, extraneural prion accumulation in SLOs is not strictly dependent on the presence of mature FDCs. Although prion titers remain below detection in spleens of i.p.-inoculated *TNFR1^−/−^* and *TNFα^−/−^* mice, PrP^Sc^ levels and prion infectivity in *TNFR1^−/−^* and *TNFα^−/−^* lymph nodes are only marginally reduced compared to *TNFR1^−/−^*, *LTβR^−/−^*, *LTα^−/−^*, or *LTβ^−/−^* spleens [30], [31]. Furthermore, *TNFR1^−/−^* and *TNFα^−/−^* mice succumb to terminal disease upon i.p. prion inoculation at a noticeably higher rate than lymphotoxin signaling-deficient mice, indicating that prions are still effectively transmitted to the CNS in the absence of TNFR1 signaling. Since *TNFR1^−/−^* lymph nodes are devoid of detectable mature FDCs, this implies that other undefined cell types may also be required for prions to colonize SLOs. However, lymph nodes are either absent or profoundly disrupted in *LTβR^−/−^*, *LTα^−/−^*, and *LTβ^−/−^* mice compared to *TNFR1^−/−^* and *TNFα^−/−^*, making it difficult to formally conclude that LTβR signaling is specifically required for prion accumulation in lymph nodes while TNFR1 is not. To determine whether continuous LTβR signaling is required for prions to accumulate in *TNFR1^−/−^* lymph nodes, we investigated the ability of prions to colonize SLOs of prion-infected *TNFR1^−/−^* mice treated with an LTβR-Ig blocking antibody.

## Results

### Prion accumulation in *TNFR1^−/−^* lymph nodes is LTβR signaling-dependent

**Figure 1:**
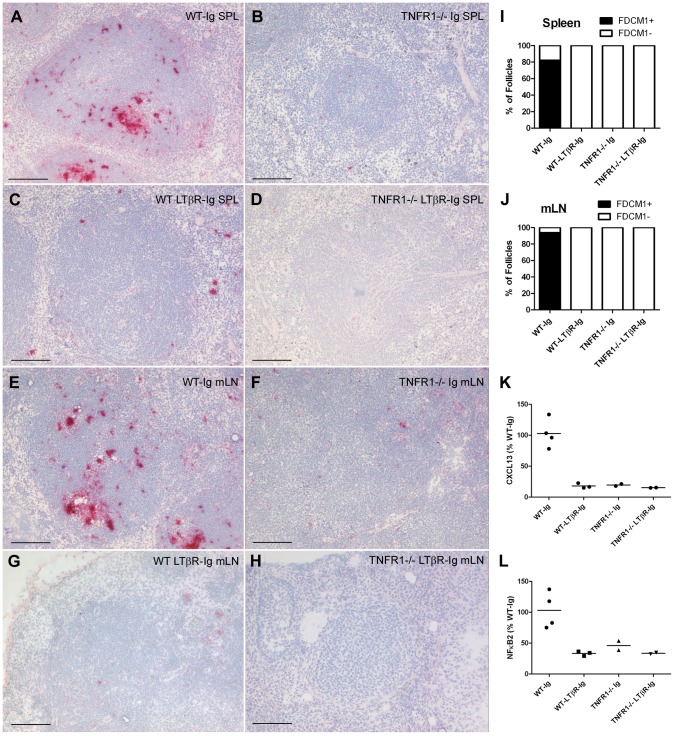
Repeated LTβR-Ig administration chronically downregulates LTβR signaling and de-differentiates FDC networks in C57BL/6 and *TNFR1^−/−^* spleens and lymph nodes. Frozen sections from spleens (**A–D**) and mesenteric lymph nodes (**E–H**) of C57BL/6 (WT) Ig-treated (**A & E**), C57BL/6 (WT) LTβR-Ig-treated (**C & G**), *TNFR1^−/−^* Ig-treated (**B & F**), or *TNFR1^−/−^* LTβR-Ig-treated (**D & H**) mice were analyzed by immunohistochemistry and developed with alkaline phosphatase for follicular dendritic cell marker 1 (FDCM1). The total number of FDCM1-positive (FDCM1+; black) or FDCM1-negative (FDCM1−; white) lymphoid follicles were scored for spleens (**I**) and mesenteric lymph nodes (mLNs; **J**) and expressed as a percentage of total follicles in each treatment group. FDCM1+ FDC networks were visible in 82% of WT Ig-treated spleen follicles (**A; I**) and 94% of WT-Ig lymph node follicles (**E; J**), whereas FDCM1+ FDCs were absent (0% of follicles) in spleens and lymph nodes from mice lacking TNFR1 and/or LTβR signaling (**B–D; F–H; I–J**). Total mRNA was isolated from spleens of mice from the indicated treatment groups and analyzed for expression of LTβR signaling targets CXCL13 (Mean ± S.E.M.: WT-Ig = 102.68±11.56, WT-LTβR-Ig = 18.05±2.30, *TNFR1^−/−^*-Ig = 19.56±1.63, and *TNFR1^−/−^*-LTβR-Ig = 15.14±0.15) (**K**) and NFκB2 (Mean ± S.E.M.: WT-Ig = 103.10±14.65, WT-LTβR-Ig = 33.30±2.10, *TNFR1^−/−^*-Ig = 46.04±7.53, and *TNFR1^−/−^*-LTβR-Ig = 33.39±1.18) (**L**) by Real Time PCR. Both CXCL13 and NFκB2 mRNA levels were reduced in all treatment groups relative to WT-Ig.

We previously showed that mice devoid of TNF signaling accumulate prion infectivity and PrP^Sc^ in lymph nodes but not in spleen, in contrast to LT signaling-deficient mice which do not accumulate prions in either spleen or lymph nodes [31]. To determine whether prion accumulation in *TNFR1^−/−^* lymph nodes was dependent on continuous LTβR signaling, we administered weekly 100 µg intraperitoneal (i.p.) injections of an LTβR immunoglobulin fusion protein (LTβR-Ig) or control pooled human immunogloblulin (Ig) to wild-type (WT) and *TNFR1^−/−^* mice to achieve sustained inhibition of LTβR signaling [32]. One week following the initial LTβR-Ig or control Ig injection, mice were inoculated intraperitoneally (i.p.) with 6 log LD_50_ RML6 prions. At 60 days post-injection (d.p.i.), spleens and mesenteric lymph nodes (mLNs) from these mice were assessed for accumulation of prion infectivity and PrP^Sc^. To confirm that LTβR-Ig treatment effectively inhibited LTβR signaling, we analyzed follicular dendritic cell marker 1 (FDCM1) immunoreactivity in spleens from WT or *TNFR1^−/−^* mice treated with either control Ig or LTβR-Ig. FDCM1 immunoreactivity was absent in spleens and mLNs from *TNFR1^−/−^*-Ig, WT-LTβR-Ig, and *TNFR1^−/−^*-LTβR-Ig spleens in contrast to WT-Ig, indicating that LTβR-dependent FDCs had de-differentiated in response to LTβR-Ig treatment (**Fig. 1A–J**). In addition, we analyzed transcriptional targets of the LTβR pathway in spleens from WT or *TNFR1^−/−^* mice treated with either control Ig or LTβR-Ig. As expected, levels of both NFκB2 (p100) and CXCL13 mRNA were reduced in *TNFR1^−/−^*-Ig, WT-LTβR-Ig, and *TNFR1^−/−^*-LTβR-Ig spleens compared to WT-Ig (**Fig. 1K & L**).

**Figure 2:**
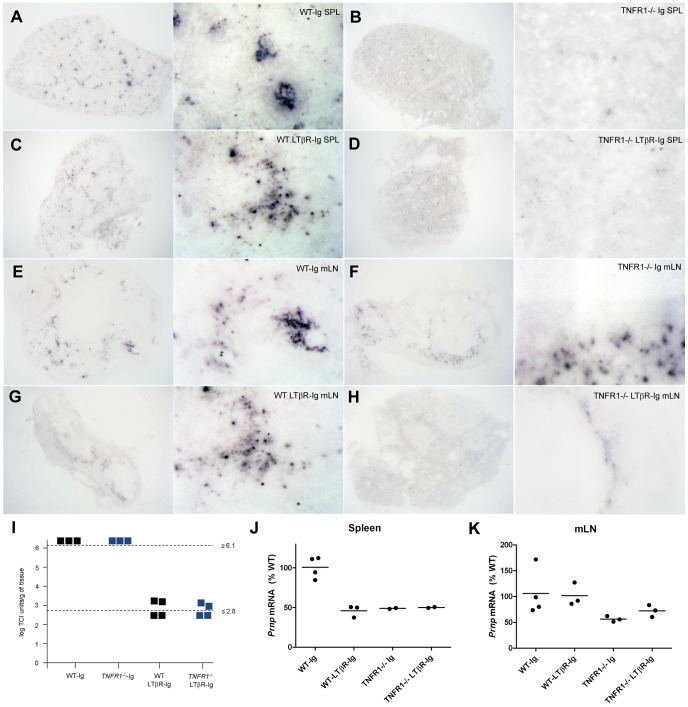
PrP^Sc^ accumulation in *TNFR1^−/−^* lymph nodes requires LTβR signaling independent of *Prnp* expression. C57BL/6 (WT) or TNFR1^−/−^ mice inoculated i.p. with 6 log LD_50_ RML6 and treated weekly with control Ig or LTβR-Ig were sacrificed at 60 d.p.i. Histoblots were performed on frozen sections from spleens (SPL; A–D) or mesenteric lymph nodes (mLN; E–H) from mice in each treatment group to visualize PrP^Sc^ deposition. Whole organs are shown in left panels, and corresponding higher resolution images for each treatment group are shown in right panels. Note that lack of TNFR1 signaling can prevent PrP^Sc^ accumulation in spleen (B) but not lymph node (F). However, blocking LTβR signaling can prevent PrP^Sc^ accumulation in TNFR1^−/−^ lymph nodes (compare F and H). Prion infectivity titers in mLN homogenates from individual TNFR1^−/−^ Ig-treated and LTβR-Ig treated mice were measured using the scrapie cell assay (I). Whereas TNFR1^−/−^-Ig mLNs all harbored ≥6.1 log TCI units/g tissue, prion infectivity in TNFR1^−/−^-LTβR-Ig mLNs was reduced by at least 2.5 log TCI units/g tissue. Total mRNA was isolated from spleens (J) or mesenteric lymph nodes (mLN; K) of mice from the indicated treatment groups and analyzed for Prnp expression by Real Time PCR (Mean ± S.E.M.: Spleen – WT-Ig = 100.69±6.74, WT-LTβR-Ig = 46.02±4.30, TNFR1^−/−^-Ig = 48.94±0.55, and TNFR1^−/−^-LTβR-Ig = 50.11±0.55; mLN – WT-Ig = 106.05±22.54, WT-LTβR-Ig = 101.69±12.88, TNFR1^−/−^-Ig = 56.43±3.13, and TNFR1^−/−^-LTβR-Ig = 72.35±6.65). Whereas spleens from WT-LTβR-Ig, TNFR1^−/−^-Ig, and TNFR1^−/−^-LTβR-Ig mice all showed decreases in Prnp expression relative to WT-Ig spleens (J), no differences could be found in Prnp expression in mLNs from mice in any treatment group (K).

Next, to determine the effect of inhibited LTβR signaling on prion accumulation in SLOs, we compared the pattern of PrP^Sc^ deposition in spleens and mLNs from prion-infected *TNFR1^−/−^*-Ig, WT-LTβR-Ig, and *TNFR1^−/−^*-LTβR-Ig mice to WT-Ig mice by histoblotting. As expected, *TNFR1^−/−^*-Ig, WT-LTβR-Ig, and *TNFR1^−/−^*-LTβR-Ig spleens accumulated less PrP^Sc^ than WT-Ig spleens (**Fig. 2A–D**), though chronic LTβR-Ig administration seemed less effective at preventing PrP^Sc^ deposition in WT spleens than genetic ablation of TNFR1 (compare **Fig. 2B** with **2C**). Since the 60 day treatment period approaches the limit of effective LTβR-Ig inhibition, this most likely reflects partial FDC re-maturation in WT SLOs near the end of the experiment (J. Browning; personal communication). Regardless, the ability of LTβR-Ig to block prion replication in WT SLOs is well-established [26], [27]. Consistent with our previous studies, mLNs from WT and *TNFR1^−/−^*-Ig-treated mice contained similar numbers of PrP^Sc^ deposits (**Fig. 2E & F**), whereas PK-resistant PrP deposits in WT-LTβR-Ig mLNs were less numerous than in WT-Ig or *TNFR1^−/−^*-Ig mLNs (Compare **Fig. 2G** with **Fig. 2E & F**). Background PrP immunoreactivity in PK-digested histoblots from non-infected wild-type spleens was negligible (**Supp. Fig. S1**).

Of note, *TNFR1^−/−^*-LTβR-Ig mLNs were devoid of PrP^Sc^ immunoreactivity (**Fig. 2H**), demonstrating that prion accumulation in lymph nodes in the absence of TNFR1 is dependent on LTβR signaling. To confirm this result quantitatively, we analyzed prion infectivity in mLNs from Ig-treated versus LTβR-Ig-treated WT and *TNFR1^−/−^* mice using the scrapie cell assay (SCA; [33], [34], [35]). Consistent with the corresponding histoblots from these mice, LTβR-Ig treatment decreased prion infectivity in WT and *TNFR1^−/−^* mLNs by 2–3 log tissue culture infectious (TCI) units per gram of tissue compared to Ig treatment (**Fig. 2I**).

To determine the effect of LTβR-Ig treatment on PrP^C^ expression in SLOs, which might explain the differential ability of *TNFR1^−/−^*-Ig versus *TNFR1^−/−^*-LTβR-Ig mLNs to accumulate prions, we measured *Prnp* mRNA levels in spleens and lymph nodes from all treatment groups by quantitative PCR. *Prnp* mRNA levels were reduced in WT-LTβR-Ig, *TNFR1^−/−^*-Ig, and *TNFR1^−/−^*-LTβR-Ig spleens compared to WT-Ig (**Fig. 2J**), most likely reflecting de-differentiation of FDCs, the primary PrP^C^-expressing cell type in spleen. In contrast, no differences in *Prnp* mRNA expression were found in mLNs from mice of different treatment groups. Of particular note, no difference in *Prnp* mRNA expression was found between *TNFR1^−/−^*-Ig and *TNFR1^−/−^*-LTβR-Ig mLNs (**Fig. 2K**). Taken together, our data indicate that *TNFR1^−/−^* lymph nodes accumulate prions in the absence of mature FDCs yet in an LTβR-dependent manner. Moreover, inhibited prion deposition in mLNs upon loss of LTβR signaling seems to be unrelated to local *Prnp* levels.

### MadCam1 expression correlates with prion infectivity in mesenteric lymph nodes

**Figure 3:**
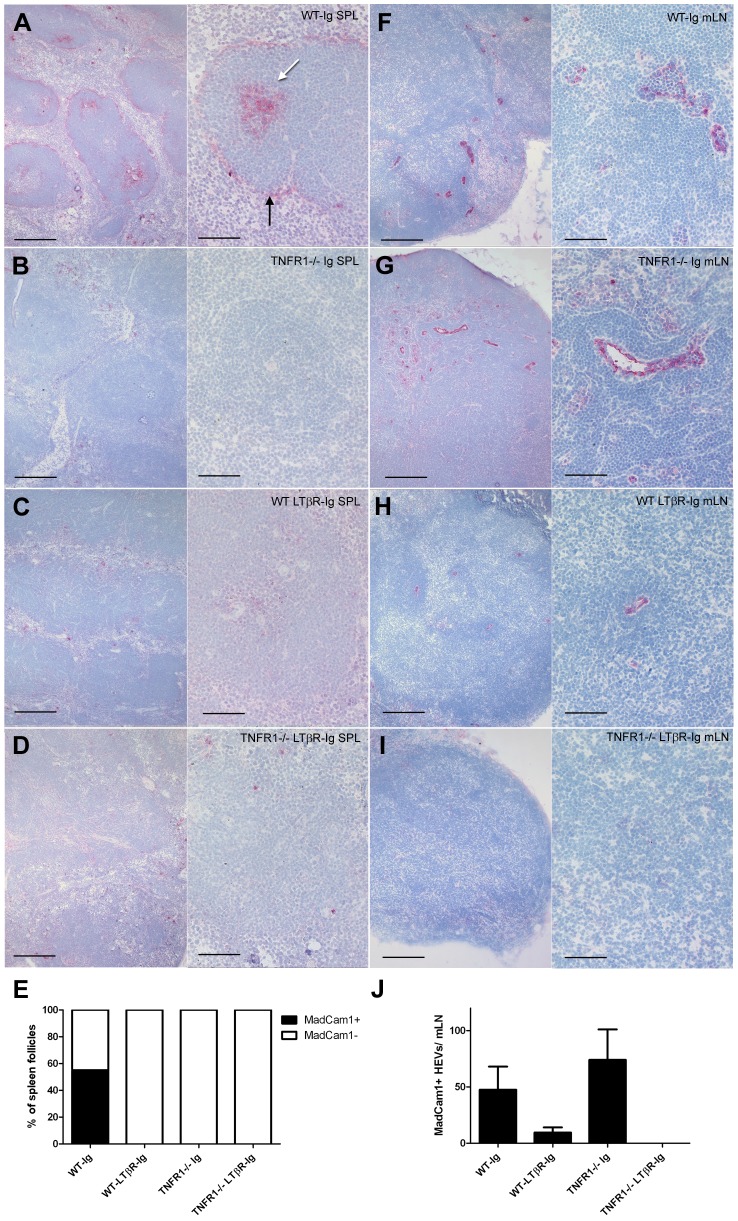
MadCam1 immunoreactivity in lymphoid tissue correlates with prion deposition. Formalin-fixed cryosections from spleens (SPL; A–D) or mesenteric lymph nodes (mLN; F–I) of C57BL/6 (WT) Ig-treated (A & F), TNFR1^−/−^ Ig-treated (B & G), C57BL/6 (WT) LTβR-Ig-treated (C & H), or TNFR1^−/−^ LTβR-Ig-treated (D & I) mice were immunostained with an antibody against the stromal cell marker, mucosal addressin cell adhesion molecule 1 (MadCam1), and visualized with alkaline phosphatase. (E) The total number of MadCam1-positive (MadCam1+; black) or MadCam1-negative (MadCam1-; white) lymphoid follicles were scored for spleens and expressed as a percentage of total follicles in each treatment group. (J) The total number of MadCam1-postive (MadCam1+) structures per mesenteric lymph node (mLN) was counted and averaged for each treatment group. WT-Ig mLNs contained 47.5±14 MadCam1+ structures, WT-LTβR-Ig = 9.5±3, TNFR1^−/−^-Ig = 74±19, and TNFR1^−/−^-LTβR-Ig = 0. MadCam1 immunoreactivity in WT-Ig spleens was localized to the marginal sinus (A; black arrow) and 55% (E) of germinal centers (A; white arrow). This staining pattern was absent (0%; E) in the spleens of mice from all other treatment groups (B,C & D). In contrast, MadCam1 immunoreactivity in WT-Ig mLNs was largely found in thick vessels (F). MadCam1 immunoreactivity was retained in TNFR1^−/−^-Ig mLNs (G) but absent in mLNs from mice treated with LTβR-Ig (H–J). Size bars: Left panels = 200 µm; Right panels = 100 µm.

**Figure 4:**
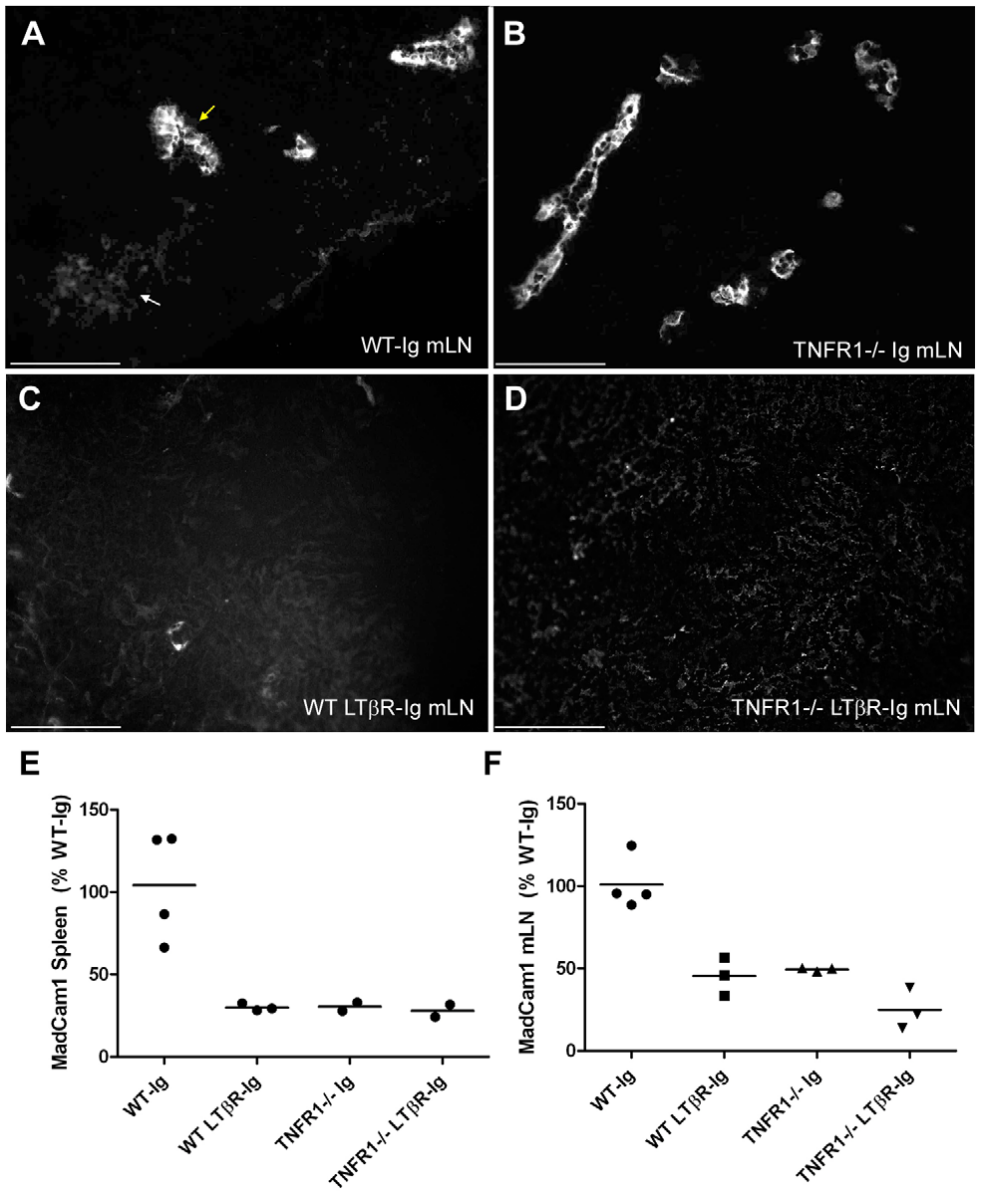
Vessel-associated MadCam1 expression in lymph nodes is preserved in the absence of TNFR1 signaling. Frozen sections from mesenteric lymph nodes (mLN) of C57BL/6 (WT) Ig-treated (A), TNFR1^−/−^ Ig-treated (B), C57BL/6 (WT) LTβR-Ig-treated (C), or TNFR1^−/−^ LTβR-Ig-treated (D) mice were analyzed by immunofluorescence with MadCam1 antibody. In WT-Ig mLNs (A), MadCam1 robustly stained thick vessels (yellow arrow), while germinal centers were weakly MadCam1-positive (white arrow). In TNFR1^−/−^-Ig mLNs (B), MadCam1-positive germinal centers were absent, while vessel-associated MadCam1 staining persisted. In contrast, both WT-LTβR-Ig and TNFR1^−/−^-LTβR-Ig mLNs were MadCam1-negative (C & D). Size bars = 100 µm. Total mRNA was isolated from spleens (E) or mesenteric lymph nodes (F) of C57BL/6 (WT) Ig-treated, C57BL/6 (WT) LTβR-Ig-treated, TNFR1^−/−^ Ig-treated, or TNFR1^−/−^ LTβR-Ig-treated mice and analyzed for MadCam1 expression by Real Time PCR (Mean ± S.E.M.: Spleen – WT-Ig = 104.21±16.56, WT-LTβR-Ig = 29.98±1.28, TNFR1^−/−^-Ig = 30.41±2.56, and TNFR1^−/−^-LTβR-Ig = 27.95±3.77; mLN – WT-Ig = 100.89±8.06, WT-LTβR-Ig = 45.40±6.75, TNFR1^−/−^-Ig = 49.42±0.63, and TNFR1^−/−^-LTβR-Ig = 24.79±7.25). MadCam1 expression was reduced in spleens of WT-LTβR-Ig-treated, TNFR1^−/−^ Ig-treated, and TNFR1^−/−^ LTβR-Ig-treated mice compared to WT-Ig. MadCam1 expression in WT-LTβR-Ig and TNFR1^−/−^ Ig mLNs was reduced compared to WT-Ig, and MadCam1 expression in TNFR1^−/−^ LTβR-Ig mLNs was reduced compared to TNFR1^−/−^-Ig.

We reasoned that prion accumulation in *TNFR1^−/−^* lymph nodes might rely on a putative LTβR signaling-dependent, TNFR1 signaling-independent cell present in lymph nodes but not in spleens. In order to identify such a cell, we screened spleens and mLNs from WT-Ig, WT-LTβR-Ig, *TNFR1^−/−^*-Ig, and *TNFR1^−/−^*-LTβR-Ig mice for a variety of hematopoietic and stromal cell markers by immunohistochemistry (IHC) whose expression correlated with prion replication ability. As previously noted, the pattern of FDCM1 immunoreactivity was consistent with PrP^Sc^ deposition in spleen, but not in mLNs (**Fig. 1A–J**). Likewise, several other markers for B cells (B220), T cells (CD3), marginal zone macrophages (MOMA-1), monocytes (F4/80), and stromal cells (CD21/35, C4, VCAM1, ICAM1) revealed no staining patterns consistent with an involvement in splenic or lymph nodal prion accumulation (**Supp. Figs S2A–H & S3A–H**). However, we identified one stromal cell marker that exhibited a staining pattern consistent with prion deposition in both spleen and mLNs: mucosal addressin cell adhesion molecule 1 (MadCam1 [36]). MadCam1 weakly stained FDC networks of both WT-Ig spleen (**Fig. 3A & E**) and lymph nodes (**Fig. 4A**), as well as the marginal sinus (MS) of spleens from WT-Ig mice (**Fig. 3A**). Although FDC and MS-associated MadCam1 immunoreactivity was absent upon loss of TNFR1 or LTβR signaling in both spleen (**Fig. 3B–E**) and lymph nodes (**Fig. 4 B–D**), MadCam1 immunoreactivity persisted in *TNFR1^−/−^*-Ig mLNs (**Fig. 3G & J** **;** **4B**). Of note, MadCam1 immunoreactivity was completely absent in mLNs from LTβR-Ig-treated WT and *TNFR1^−/−^* mice (**Fig. 3I–J** **&** **4C–D**). Furthermore, analysis of MadCam1 mRNA expression by Real Time PCR in mLNs quantitatively corroborated the MadCam1 IHC findings: MadCam1 mRNA levels were equally reduced in spleens from WT-LTβR-Ig, *TNFR1^−/−^*-Ig, and *TNFR1^−/−^*-LTβR-Ig mice, compared to WT-Ig (**Fig. 4E**). In contrast, MadCam1 mRNA levels were intermediately reduced in *TNFR1^−/−^*-Ig mLNs compared to WT-Ig, and a further reduction in MadCam1 mRNA levels was observed in *TNFR1^−/−^*-LTβR-Ig mLNs compared to *TNFR1^−/−^*-Ig (**Fig. 4F**). Thus far, our data suggested that the presence of a MadCam1-expressing cell was associated with accumulation of prions in lymph nodes but not in spleen.

### MadCam1-positive structures in *TNFR1^−/−^* lymph nodes are PrP^C^-expressing HEVs

**Figure 5:**
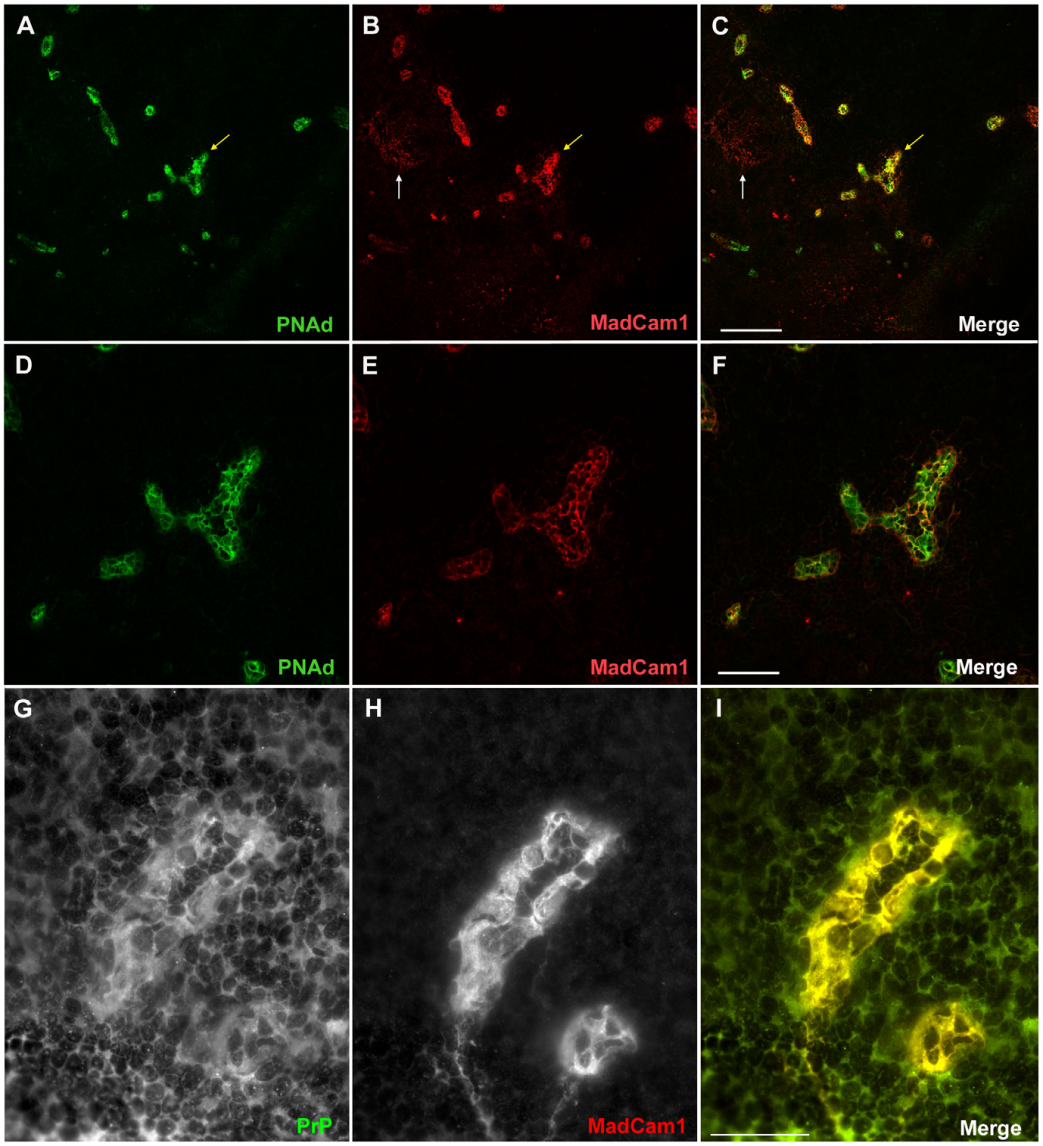
MadCam1-positive vessels in mesenteric lymph nodes are prion protein-expressing high endothelial venules. Co-immunofluorescent confocal microscopy was performed on frozen sections from WT-Ig mesenteric lymph nodes with the high endothelial venule (HEV)-specific marker, peripheral node addressin (PNAd; green; A & D) and MadCam1 (red; B & E). Merged image shown in C & F. MadCam1-positive vessels co-localized with PNAd (A,B & C; yellow arrow). Note that MadCam1-positive germinal centers were PNAd-negative (B & C; white arrow). Standard co-immunofluorescence was performed on frozen sections from WT-Ig mesenteric lymph nodes with MadCam1 (red; G) and an antibody directed against the C-terminus of the prion protein (PrP; green; H; POM1; [62]). MadCam1-positive HEVs were also PrP-positive (I). Size bars in A–C = 150 µm. Size bars in D–F = 50 µm. Size bars in G–I = 20 µm.

MadCam1 immunoreactivity in *TNFR1^−/−^*-Ig mLNs was restricted to thick vessels (**Fig. 3G** **&** **4B**) that were absent from *TNFR1^−/−^*-LTβR-Ig mLNs (**Fig. 3I** **&** **Fig. 4D**), as well as isotype controls (**Supp. Fig. S4B**), and were morphologically distinct from MadCam1-positive FDC networks found in WT-Ig spleens (**Fig. 3A**) and mLNs (**Fig. 4A**). These MadCam1-positive structures were morphologically consistent with high endothelial venules (HEVs) responsible for controlling lymphocyte entry into LNs, Peyer's patches (PPs), and other SLOs, with the exception of spleen [37]. Co-localization of Madcam1-positive vessels with peripheral node addressin (PNAd; a.k.a. MECA-79), a specific marker of HEVs [38], confirmed that these structures in *TNFR1^−/−^*-Ig mLNs were HEVs (**Fig. 5A–F**), whereas MadCam1-positive FDC networks in WT-Ig mLNs (**Fig. 5A–C**) and isotype controls (**Supp. Fig. S4A**) were PNAd-negative. To gather evidence that HEVs could be potential sites of prion replication, we analyzed PrP^C^ immunoreactivity in HEVs of WT-Ig mLNs. Co-immunofluorescent (co-IF) staining of WT-Ig mLNs with MadCam1 and PrP^C^ antibodies confirmed that PrP^C^ immunoreactivity was present in HEVs (**Fig. 5G–I**) and absent from isotype controls (**Supp. Fig. S4C**). Thus, HEVs fulfill at least one requirement of a prion replicating tissue - PrP^C^ expression.

### PrP^Sc^ localizes to HEVs in *TNFR1^−/−^* lymph nodes

**Figure 6:**
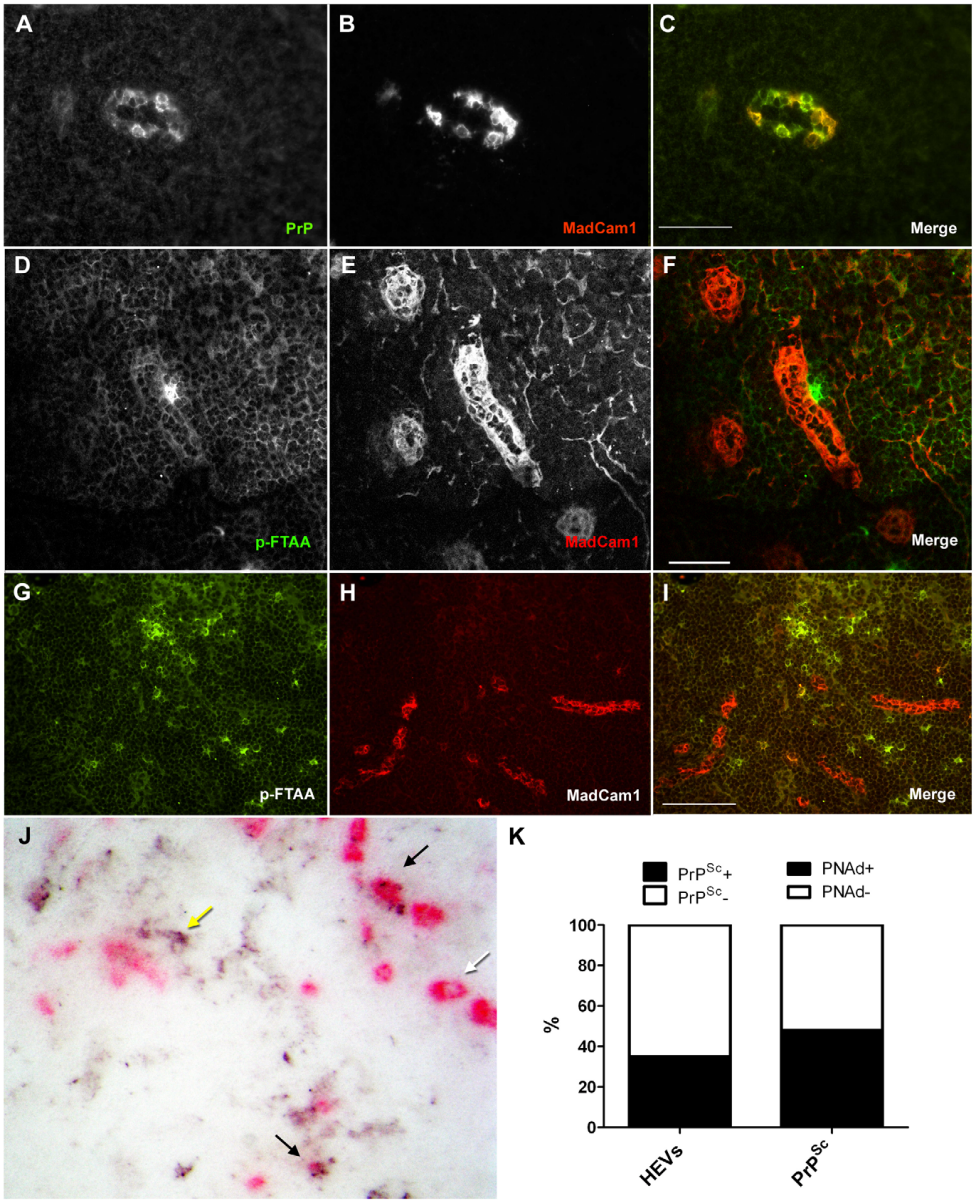
PrP^Sc^ is present both in and around MadCam1-positive HEVs in *TNFR1^−/−^*-Ig mesenteric lymph nodes. TNFR1^−/−^ mice inoculated i.p. with 6 log LD_50_ RML6 and treated weekly with control Ig were sacrificed at 60 d.p.i. Immunofluorescence (A–I) and histoblots (J & K) were then performed on frozen sections from prion-infected TNFR1^−/−^-Ig mesenteric lymph nodes. Co-IF with anti-serum (XN) against PrP (green; A) and MadCam1 (red; B) showed points of intense PrP immunoreactivity localized to HEVs (C). Confocal co-IF with the amyloid-binding dye, p-FTAA (green; D) and MadCam1 (red; E,F) revealed some points of PrP^Sc^ association with HEVs (F); however much of the PrP^Sc^ was present outside of HEVs (I). Histoblots pre-stained with PNAd antibody and developed with AP (pink; J) also revealed some prion-infected HEVs (black arrows), some non-infected HEVs (white arrow), and some PrP^Sc^ deposits that were not HEV-associated (yellow arrow). (K) Total numbers of PNAd-positive HEVs in histoblot co-stains were counted and scored as PrP^Sc^-positive (PrP^Sc^+; black) or PrP^Sc^-negative (PrP^Sc^+; white), and total PrP^Sc^ deposits were counted and scored as PNAd-positive (PNAd+; black) or PNAd-negative (PNAd; white). 35% of HEVs were PrP^Sc^-positive, and 58% of PrP^Sc^ deposits were PNAd-positive. Size bars in A–F = 50 µm. Size bars in G–I = 100 µm.

To determine whether prions were localized to HEVs in mLNs, we performed co-IF with PrP^C^ and MadCam1 antibodies in prion-infected *TNFR1^−/−^*-Ig mLNs. Prion-infected *TNFR1^−/−^*-Ig mLNs contained areas of intense PrP-positive deposits which were not detectable in non-infected *TNFR1^−/−^*-Ig mLNs (data not shown; [31]). Of note, many of these PrP^C^-positive areas localized to MadCam1-postive vessels in prion-infected *TNFR1^−/−^*-Ig mLNs, indicating that HEVs are probable sites of PrP^Sc^ localization in infected lymph nodes (**Fig. 6A–C**). However, PrP immunostaining of prion-infected tissue cannot reliably distinguish between PrP^Sc^ deposits and sites of high PrP^C^ expression, and HEVs expressed relatively high levels of PrP^C^ in uninfected mLNs (**Fig. 5G–I**).

To develop an independent method of distinguishing PrP^Sc^ from PrP^C^ in lymphoid organs, we tested the ability of a series of fluorescent amyloid-binding dyes known as luminescent conjugated polymers (LCPs [39], [40], [41], [42]) to stain PrP^Sc^ deposits in spleen and lymph node. LCPs were previously shown to recognize PrP^Sc^ in brain [43]. One LCP, pentameric formic thiophene acetic acid (p-FTAA; [44]), stained PrP-positive FDC networks of prion-infected spleens from WT mice (**Supp. Fig. S5**). In contrast, no immunofluorescence could be detected with p-FTAA in spleens and lymph nodes from uninfected mice (**Supp. Fig. S6**). Using this method, points of PrP^Sc^/MadCam1 co-localization could be reliably identified in prion-infected *TNFR1^−/−^* lymph nodes, which again indicated that a proportion of PrP^Sc^ localizes to HEVs in *TNFR1^−/−^* mLNs (**Fig. 6D–F**). However, at lower magnification we also noted that a number of PrP^Sc^ deposits were located outside of HEVs (**Fig. 6G–I**). To confirm this observation and to analyze the tissue-wide distribution of PrP^Sc^ deposits relative to HEVs in prion-infected *TNFR1^−/−^*-Ig mLNs, we performed histoblot co-stains with PNAd antibody, which is highly immunoreactive to HEVs and can be visualized even after proteinase K digestion (**Supp. Fig. S7**). This analysis confirmed that some PrP^Sc^ deposits were localized to HEVs in *TNFR1^−/−^*-Ig mLNs. However, PrP^Sc^ deposits were also present outside of HEVs (**Fig. 6J–K**), which is consistent with our previous study showing that strong PrP immunoreactivity in prion-infected *TNFR1^−/−^* mLNs can also be found in other cell types (i.e. macrophages [31]). Since HEVs could potentially serve as entry portals for prion-harboring lymphocytes, and it was recently reported that neighboring dendritic cells (DCs) are responsible for HEV differentiation [45], we also performed PrP co-immunofluorescence on prion-infected *TNFR1^−/−^*-Ig mLNs using the DC marker, CD11c, to determine whether DCs in the vicinity of HEVs might also contain PrP^Sc^. However, no overlap of PrP and CD11c immunoreactivity in prion-infected *TNFR1^−/−^*-Ig mLNs was identified (**Supp Fig. S8**).

## Discussion

The means by which prions evade the immune system's numerous defense mechanisms and finally transmigrate to, and selectively damage, neurons of the CNS has been the subject of scientific scrutiny for two decades. A number of studies have implicated FDCs in the germinal centers of SLOs as the primary reservoirs of prions prior to neuroinvasion [4], [12], [13], [14], [15], [16], [17]. Yet the ability of *TNFR1^−/−^* mLNs to accumulate prions with a minimal loss of infectivity compared to WT mLNs presents an apparent paradox, since FDC maintenance depends on TNFR1 signaling [21], [22], [23].

Here we have established that lymph nodal prion accumulation in the absence of TNFR1 signaling is LTβR signaling-dependent. Crucially, transient loss of LTβR signaling was sufficient to block TNFR1-independent prion accumulation in lymph nodes, indicating that prion accumulation in lymph nodes specifically requires LTβR signaling and is not simply prevented by general developmental defects or architectural disruptions caused by lack of LTβR signaling in *LTβ^−/−^* lymph nodes.

We previously showed that intense PrP immunoreactivity was localized to macrophages in prion-infected *TNFR1^−/−^* mLNs [31], and others have reported that PK-resistant PrP is localized to macrophages in spleens with PrP^C^-deficient FDCs [46], indicating that macrophages serve as alternative sites of prion accumulation in the absence of PrP^C^-expressing FDCs. How this phenomenon was mechanistically linked to LTβR signaling and the pattern of prion accumulation in lymph nodes was initially unclear, since most macrophage populations were preserved in the absence of both TNFR1 and LTβR signaling [31]. Here, we discovered that loss of LTβR signaling in mLNs was also correlated with the dedifferentiation of HEVs – the primary point of entry for lymphocytes into lymph nodes and a likely determinant in the ability of prions to colonize lymph nodes.

Consistent with the pattern of prion accumulation in spleens and lymph nodes of mice lacking TNF and/or LT signaling components, HEVs exist in lymph nodes and other SLOs but not spleens [37], and the maintenance of HEV architecture is TNFR1 signaling-independent yet LTβR signaling-dependent [47]. Moreover, we identified sites of PrP^Sc^-HEV overlap in *TNFR1^−/−^*-Ig mLNs, indicating that HEVs might replicate prions and/or serve as points of entry for prions or prion-harboring lymphocytes. FDC-deficient mice can succumb to scrapie even in the absence of detectable splenic prion titers [24]. In light of our current results, this phenomenon is most likely explained by HEV-dependent entry and accumulation of prions in lymph nodes and other HEV-containing SLOs, such as Peyer's patches.

HEVs can also form ectopically in certain chronic inflammatory conditions. Prion accumulation at sites of chronic inflammation has previously been observed, but in most cases this could be attributed to local formation of FDC networks [48], [49], [50]. However, we also previously reported LTβR-dependent prion colonization of granulomas in the absence of FDC markers [51]. Since ectopic HEV formation without any evidence of FDC networks has previously been reported in certain inflammatory conditions [52], ectopic HEV formation might be a source of prion replication and/or uptake in granulomas, as well.

Of note, LTα/β signaling to HEVs differs from LTα/β signaling to FDCs. Though HEVs express LTβR [53], HEVs persist in the absence of B cells [47], in contrast to FDCs which rely on B cells to provide LTα/β ligand [19], [20]. This indicates that the LTα/β signal to HEVs emanates from another cell type. Until recently the cell type providing LTα/β to HEVs was not known; however a recent publication reports that dendritic cells (DCs) may be the source of LTα/β signaling to HEVs [45]. If HEV differentiation is indeed B cell-independent, this may explain why prion neuroinvasion can occur in the absence of B cells in some cases, since LTβR signaling to HEVs and hence the ability of prions to enter and accumulate in lymph nodes would be preserved [24], [54].

Do prions actually replicate in *TNFR1^−/−^* lymph nodes, or do they simply accumulate? Accumulation seems likely, as the only cell type in SLOs known to replicate prions are FDCs, and prion replication in macrophage populations was not observed [46]. In any case, experimental evidence suggests that prion accumulation in SLOs is sufficient for prions to invade the CNS [55], [56], [57]. Moreover, deletion of complement factors dramatically impairs prion accumulation in SLOs and subsequent neuroinvasion without affecting PrP^C^ levels [58], indicating that the ability of SLOs to physically capture prions is indeed critical for the development of downstream pathology.

Curiously, we previously showed that PrP^C^ expression is required in either the stromal or the hematopoietic compartment for lymph nodes to accumulate prion infectivity [31], which implies that prion replication can occur in both compartments in lymph nodes. However, an alternative explanation is that a hematopoietic cell is required for delivery of prions to a “trapping” cell within the lymph node, and PrP^C^ expression on the hematopoietic cell mediates efficient uptake of PrP^Sc^ in the bloodstream. Hence, HEVs may facilitate the selective uptake and accumulation of prions or prion-containing lymphocytes into lymph nodes, rather than serving as sites of *bona fide* prion replication. Based on both current and past findings, it is likely that the “trapping” cells in lymph nodes are endothelial cells in HEVs (HEVECs), and the hematopoietic delivery cells are macrophages. Once prions or prion-harboring cells have successfully invaded mLNs through HEVs, they may be transported *via* conduits to FDCs [59] under normal conditions. However, in the absence of mature FDCs, prions may remain in or be transferred to macrophages.

## Materials and Methods

### Animals, treatments & ethical statement

*TNFR1^−/−^* mice carrying a targeted deletion of exons 2, 3, and part of exon 4 of the tumor necrosis factor receptor 1 (TNFR1) open reading frame [60] were maintained on a C57BL/6 background in-house. C57BL/6 wild-type control mice were purchased from Harlan Laboratories and bred in-house. Murine LTβR-murine IgG1 (LTβR-Ig) and MOPC21 mouse immunoglobulin control were obtained from Biogen Idec. For analysis of prion-infected tissues (Figs 2A–I, 6 & Supp. Figs S5, S7 & S8), *TNFR1^−/−^* or C57BL/6 mice were injected intraperitoneally (i.p.) with 100 µg LTβR-Ig or MOPC21 (n = 3–4/group). One week later, mice were inoculated i.p. with 100 µL 6 log LD_50_ Rocky Mountain Laboratory mouse-adapted prion strain [61], passage 6 (RML6). Mice were then boostered weekly with 100 µg LTβR-Ig or MOPC21 until 60 days post-inoculation (d.p.i), at which point mice were sacrificed. For analysis of uninfected tissue (Figs 1, 2J–K, 3, 4, 5 & Supp. Figs S1, S2, S3, S4 & S6), *TNFR1^−/−^* or C57BL/6 mice received 2 weekly injections of LTβR-Ig or MOPC21 and were sacrificed 3 weeks following the initial injection (n = 3–4/group). Spleens and lymph nodes from prion-infected and uninfected mice were either flash frozen for scrapie cell assays and gene expression analysis or frozen in 1x Hank's balanced salt solution for immunohistochemistry. Tissues were stored at −80°C until analysis. All animal experiments were carried out in strict accordance with the rules and regulations for the Protection of Animal Rights (Tierschutzverordnung) of the Swiss Bundesamt für Veterinärwesen and were pre-approved by the Animal Welfare Committee of the Canton of Zürich. Animal permit # 130/2008.

### RML inoculum

CD1 wild-type mice were inoculated i.c. with 30 µL 1% (w/v) RML-5-infected brain homogenate and sacrificed at terminal stage disease. Brains were flash frozen in liquid nitrogen and stored at −80°C. Brains were then homogenized in sterile 0.32 M sucrose in 1x PBS (20% w/v) using a Ribolyser (Hybaid, Catalys). Subsequent dilutions of inoculum were performed in sterile 5% (w/v) bovine serum albumin (BSA) in 1x PBS. For titer determinations, serial dilutions of RML6 were inoculated i.c. into indicator mice, and LD_50_ was defined as the dilution that induced a 50% attack rate.

### Real time PCR

Frozen spleens and lymph nodes were homogenized in Qiazol (10% w/v) using a TissueLyser, and total mRNA was isolated using an RNeasy Mini kit, according to the manufacturer's instructions (Qiagen). 1 µg mRNA from each sample was used for first-strand cDNA synthesis using random hexamers from a Superscript II kit (Invitrogen). ∼100 ng cDNA, 500 nM primers, and 1x Faststart SYBR Green reaction mixture (Roche) in 25 µL reaction volumes were used for Real Time PCR amplification of target sequences from spleen and lymph nodes normalized to GAPDH using an ABI 7900HT (Applied Biosystems). (See **Table 1** for list of primer sequences.) Reactions from each tissue were performed in triplicate and averaged. Samples with a triplicate variability exceeding 10% were eliminated from further analysis. Amplification data was analyzed using the relative quantification method (RQ) with WT-Ig samples serving as the calibrator values. RQ values were calculated and averaged for each sample. Average values depicted in the graphs represent the mean value of single spleens or lymph nodes from each individual mouse from each treatment group. N = 2–4 mice per group, depending on the specific tissue and treatment group.

**Table 1:** Primer sequences used for Real Time PCR analysis.

Transcript	Primer Sequence
mCXCL13	F – 5′ - TGGCCAGCTGCCTCTCTC – 3′
	R – 5′ - TTGAAATCACTCCAGAACACCTACA - 3
mNFkB2	F – 5′ - GCA GAG AATGAGGAGCCTCTGTG – 3′
	R – 5′ - GCCTCGGAAGTTTCTTTGGGTAT – 3′
mPrnp	F – 5′ - GCC AGT GGA TCA GTA CAG CA – 3′
	R – 5′ – ATCCCACGATCAGGAAGATG – 3′
mMadCam1	F – 5′ - CCT ACA TCC TGA CCT CAT CAA GT – 3′
	R – 5′ - AGA GCT CAG AGT CCT AGG GCT AA – 3′
mGAPDH	F – 5′ – CCACCCCAGCAAGGAGACT – 3′
	R – 5′ – GAAATTGTGAGGGAGATGCT – 3′

### Histoblots

10 µM frozen sections were transferred to nitrocellulose pre-soaked in 1x Tris-buffered saline with Tween 20 (TBST; 50 mM Tris-HCl, pH = 7.8, 150 mM NaCl, 0.1% Tween 20) and air-dried. Membranes were washed in 1x TBST for 1 hr. and then digested with proteinase K (Roche, 20 µg/mL) diluted in digestion buffer (10 mM Tris-HCl, pH = 7.8, 100 mM NaCl, 0.1% (v/v) Brij 35) at 37°C for 4 hrs. Membranes were then washed in TBST, incubated in denaturing solution (10 mM Tris-HCl, pH = 7.8, 3 M guanidine thiocyanate) for 10 min, washed in 1x TBST, blocked in 5% dried milk in TBST for 1 hr, and then probed with 0.1 µg/mL POM1 [62] diluted in 1% milk in 1x TBST overnight at 4°C. Membranes were then washed in 1x TBST, blocked in 1% milk in 1x TBST, and then incubated with 1 µg/mL alkaline phosphatase (AP)-conjugated goat anti-mouse secondary antibody (Dako) for 1 hr. Membranes were then washed in 1x TBST, 10 min. in B3 (100 mM Tris, 100 mM NaCl, 100 mM MgCl2, pH = 9.5), and then developed for 40 min. with BCIP/NBT (Roche). Histoblot sections were then washed in distilled water, dried, and imaged using an Olympus SZX12 stereomicroscope. Uninfected spleens from wild-type mice served as negative controls for background PrP immunoreactivity (Supp. Fig. S1).

### Scrapie cell assay in endpoint format

Scrapie-susceptible neuroblastoma cells (subclone N2aPK1, [33]) were incubated with uninfected brain homogenate, defined titers of RML6-infected brain homogenate, or 10^−3^ to 10^−6^ dilutions of mesenteric lymph node homogenate from WT-Ig, WT-LTβR-Ig, *TNFR1^−/−^*-Ig, or *TNFR1^−/−^*-LTβR-Ig for 3 days. Infected N2aPK1 cells were passaged 1∶3 three times every 2 days, and then 1∶10 four times every 3 days. After reaching confluence, 2×10^4^ cells from each well were filtered onto the membrane of an ELISPOT plate (Millipore; MultiScreenHTS filter plates with Immobilon-P PVDF membrane) and denatured with 0.5 µg/mL proteinase K (PK). Individual prion-infected cells were immunodetected with POM1. Wells were scored positive if the spot number exceeded mean background values, determined as three times the standard deviation of the uninfected control. In this experiment, an ELISPOT membrane with ≥3 PrP^Sc+^ colonies was regarded as infected. From the proportion of negative to total wells, the number of tissue culture infectious units per mL was calculated with the Poisson equation. In two independent experiments, a 10^−8^ dilution of a standard inoculum (brain homogenate from a terminally scrapie-sick mouse) yielded 11/24 or 12/24 positive wells, corresponding to a titer of ∼8.3 log tissue culture infectivity (TCI) units/g of brain tissue for the initial inoculum. The sensitivity threshold was calculated to be 2.8 log TCI units/g of brain tissue.

### Immunohistochemistry

7 µM frozen sections on glass coverslips (Thermofisher) were dried for several hours at room temp, fixed in 4% formalin for 2 min, 50% acetone for 2 min, 100% acetone for 2 min, and 50% acetone for 2 min. Sections were then washed in 1x PBS, then 1x PBS+ 0.05–0.1% Tween 20, and blocked for 1 hr. in SuperBlock (Pierce). Sections were then incubated overnight at 4°C in primary antibody (see **Table 2** for antibodies and dilutions) diluted in 1∶10 SuperBlock. For vessel stains, isotype controls (rat IgM for PNAd, Pharmingen # 553941; rat IgG2a for MadCam1, eBioscience # 14-4321; and mouse IgG1 for PrP, Sigma # 15381) were performed using equivalent concentrations to the corresponding primary antibodies (Supp. Fig. S4). Sections were then washed in 1x PBS, followed by 1x PBST, then incubated with 0.2–0.4 µg/mL Alexa Fluor secondary antibody (Invitrogen) diluted in 1x PBST (for immunofluorescence [IF]) or 5.3 µg/mL unconjugated goat anti-rat secondary antibody (Caltag Laboratories) or goat anti-mouse (Jackson Immunoresearch) diluted in 1∶10 SuperBlock for 1 hr. at room temperature (for light microscopy [LM]). Sections were then washed in 1x PBS followed by 1x PBST and incubated in 7.5 µg/mL AP-conjugated donkey anti-goat tertiary antibody (Jackson Immunoresearch) in 1x PBST for 1 hr for LM. Sections were then washed and developed with Fast Red (Sigma) staining kit and counter-stained with hematoxylin & eosin (H&E) for LM. Sections were then mounted (fluorescent mounting medium for IF or aqueous mounting medium for LM; Dako) and coverslipped. Sections were imaged using an Olympus BX61TRF fluorescent microscope, a Zeiss Axiophot light microscope, or a Leica SP5 confocal microscope (where indicated).

**Table 2:** Antibodies used for immunohistochemistry and immunofluorescence.

Antibody	Product No.	Source	Working Conc.
MadCam1	553805	BD Pharmingen	20 µg/mL
POM1 mouse monoclonal	N/A	Polymenidou, 2008	1 µg/mL
PrP (XN) rabbit antiserum	N/A*	Montrasio, 2000	1∶1000
FDCM1	551320	BD Pharmingen	6.25 µg/mL
CD21/35	553817	BD Pharmingen	5 µg/mL
F4/80	MCA497R	Serotec	10 µg/mL
B220	553084	BD Pharmingen	2.5 µg/mL
FITC-MOMA-1	MCA947F	Serotec	2 µg/mL
C4	Abcam	ab11863	1 µg/mL
VCAM1	MCA1229	Serotec	10 µg/mL
ICAM1	MCA818	Serotec	5 µg/mL
PE-CD11c	553802	BD Pharmingen	4 µg/mL
PNAd	553863	BD Pharmingen	5 µg/mL
CD3 supernatant	RM-9107-S1	Thermoscientific	1∶50

* Immunogen = full-length recombinant mouse PrP^C^.

### Image quantification

**FDCM1**: Boundaries of lymphoid follicles were identified by H&E counterstaining in LM images of AP-developed spleens and lymph nodes immunostained with FDCM1. Follicles containing FDCM1-stained germinal centers were classified as “FDCM1-positive,” whereas follicles devoid of FDCM1 staining were classified as “FDCM1-negative.” The total number of FDCM1-postive or negative follicles per treatment group was expressed as a percentage of the total follicles. A total of 55 follicles were scored for WT-Ig spleens, 39 for WT-LTβR-Ig spleens, 111 for *TNFR1^−/−^*-Ig spleens, 27 for *TNFR1^−/−^*-LTβR-Ig spleens, 19 for WT-Ig mLNs, 10 for WT-LTβR-Ig mLNs, 13 for *TNFR1^−/−^*-Ig mLNs, and 10 for *TNFR1^−/−^*-LTβR-Ig mLNs. **MadCam1**: Boundaries of lymphoid follicles were identified by H&E counterstaining in LM images of AP-developed spleens immunostained with MadCam1. Follicles containing MadCam1-stained germinal centers were classified as “MadCam1-positive,” whereas follicles devoid of MadCam1 staining were classified as “MadCam1-negative.” The total number of MadCam1-postive or negative follicles per treatment group was expressed as a percentage of the total follicles. For mLNs, the total number of MadCam1-positive vessels was scored per organ and averaged. A total of 80 follicles were scored for WT-Ig spleens, 37 for WT-LTβR-Ig spleens, 137 for *TNFR1^−/−^*-Ig spleens, 39 for *TNFR1^−/−^*-LTβR-Ig spleens, n = 2 mLNs for all treatment groups. **PNAd/PrP^Sc^ co-stains**: Total numbers of HEVs and PrP^Sc^ deposits were counted on PNAd pre-stained histoblots from *TNFR1^−/−^*-Ig mLNs. HEVs that overlapped with PrP^Sc^ deposits were scored as “PrP^Sc^-postive,” whereas HEVs with no PrP^Sc^ were scored as “PrP^Sc^-negative.” Likewise, PrP^Sc^ deposits that overlapped with HEVs were scored as “PNAd-positive,” whereas PrP^Sc^ deposits that were not associated with HEVs were scored as “PNAd-negative.” For HEVs, values represent the percentage of total HEVs that were either PrP^Sc^-positive (black) or negative (white). For PrP^Sc^ deposits, values represent the percentage of total PrP^Sc^ deposits that were associated with HEVs (black) or not (white). A total of 82 HEVs and 112 PrP^Sc^ deposits were scored.

### Pentameric formic thiophene acetic acid (pFTAA) co-staining

7 µM frozen sections on glass coverslips were dried and then fixed in pre-chilled 100% acetone or ethanol at −20°C for 10 min. Sections were dried for 1 min., re-hydrated in 1x PBS for 10 min., blocked in SuperBlock, and then incubated in 20 µg/mL MadCam1 or 6.25 µg/mL FDCM1 in 1x PBS overnight at 4°C. Sections were then washed in 1x PBS and incubated with 0.2–2 µg/mL Alexa Fluor 594-conjugated goat anti-rat secondary antibody for 1 hr. Sections were then washed in 1x PBS and incubated with 30 µM pentameric formic thiophene acetic acid (p-FTAA; [44]) in 1x PBS for 30 min. Sections were then washed in 1x PBS, mounted with fluorescent mounting medium (Dako), coverslipped, and imaged using an Olympus BX61TRF fluorescent microscope. Sections from uninfected WT spleens were used as negative controls for non-specific pFTAA staining (Supp. Fig. S6).

### Histoblot co-stains

*TNFR1^−/−^*-Ig mesenteric lymph node sections (10 µm) on nitrocellulose were soaked in 1x TBST for 1 hr., blocked in 5% (w/v) milk in 1x TBST for 1 hr., and then probed with 0.5 µg/mL PNAd antibody diluted in 1% (w/v) milk in 1x TBST overnight at 4°C. Histoblots were then washed in 1x TBST and probed with 0.5 µg/mL AP-conjugated goat anti-rat (Biosource # ARI3405) secondary antibody in 1% (w/v) milk in 1x TBST. Histoblots were then washed in 1x TBST and developed with Fast Red (Sigma) for 20 min. Histoblots were then washed in 1x TBST, digested with 20 µg/mL PK for 4 hrs at 37°C, washed and further processed as described above for standard histoblots.

## Supporting Information

Figure S1 **Low basal PrP immunoreactivity in histoblots from spleens of uninfected wild-type mice.** Histoblots were performed on frozen sections from uninfected C57BL/6 (WT) spleens to determine the background level of PrP^Sc^ staining in tissue devoid of PrP^Sc^. (A) Whole organ. (B) High resolution image.
(TIF)

Figure S2 **Common stromal markers in lymphoid organs do not correlate with prion deposition.** Frozen sections from spleens (A, C, E & G) and mesenteric lymph nodes (B, D, F & H) of C57BL/6 (WT) Ig-treated, C57BL/6 (WT) LTβR-Ig-treated, TNFR1^−/−^ Ig-treated, or TNFR1^−/−^ LTβR-Ig-treated mice were analyzed by immunohistochemistry and developed with alkaline phosphatase (A & B) or immunofluorescence (C–H) for CD21/35 (A & B), complement factor C4 (C & D), vascular cell adhesion molecule 1 (VCAM1; E & F), and intercellular adhesion molecule 1 (ICAM1; G & H). Size bars in A & C = 200 µm; C–H = 100 µm.
(TIF)

Figure S3 **Common hematopoietic markers in lymphoid organs do not correlate with prion deposition.** Frozen sections from spleens (A, C, E & G) and mesenteric lymph nodes (B, D, F & H) from C57BL/6 (WT) Ig-treated, C57BL/6 (WT) LTβR-Ig-treated, TNFR1^−/−^ Ig-treated, or TNFR1^−/−^ LTβR-Ig-treated mice were analyzed by immunohistochemistry and developed with alkaline phosphatase (A–D) or immunofluorescence (E–H) for macrophages (F4/80; A & B), B-cells (B-cells; C & D), metallophilic macrophages (MOMA-1; E & F), and T-cells (CD3; G & H). Size bars in A & C = 100 µm; B & D = 200 µm; E–H = 100 µm.
(TIF)

Figure S4 **Neither rat nor mouse isotype controls immunoreact with mesenteric lymph node vessels or follicles.** Frozen sections from mesenteric lymph nodes of C57BL/6 (WT) mice were immunostained with rat IgM (A), rat IgG2a (B), or mouse IgG1 (C) isotype controls and developed with alkaline phosphatase. No specific staining of vessels or follicles was observed. Size bars = 200 µm.
(TIF)

Figure S5 **Pentameric formic thiophene acetic acid detects prion-infected FDC networks in spleens.** Frozen sections from spleens of prion-infected C57BL/6 mice were analyzed by standard (A–C; G–I) or confocal (D–F; J–L) immunofluorescence with follicular dendritic cell marker 1 (FDCM1; orange; A & red; D) or prion protein antibody POM1 (PrP; red; G & J) and pentameric formic thiophene acetic acid (p-FTAA; green; B, E, H & K). p-FTAA co-localizes with PrP-positive (I & L; overlay) FDC networks (C & F; overlay) of prion-infected mice. Size bars in C & I = 100 µm; size bar in F = 60 µM; size bar in L = 40 µm.
(TIF)

Figure S6 **Pentameric formic thiophene acetic acid does not detect PrP-positive FDC networks in uninfected spleens.** Frozen sections from spleens of uninfected C57BL/6 mice were analyzed by standard immunofluorescence with follicular dendritic cell marker 1 (FDCM1; red; A) or prion protein antibody POM1 (PrP; red; D) and pentameric formic thiophene acetic acid (p-FTAA; green; B & E). No p-FTAA staining was detected in PrP^C^-positive (C; overlay) FDC networks (F; overlay) of uninfected mice. Size bars = 100 µm.
(TIF)

Figure S7 **Tissue-wide analysis of PNAd pre-stained histoblots from prion-infected** ***TNFR1^−/−^*** **-Ig mLNs show that a proportion of PrP^Sc^ deposits co-localize to HEVs.** Histoblots of mesenteric lymph nodes from TNFR1^−/−^ mice inoculated i.p. with 6 log LD_50_ RML6, treated weekly with control Ig, and sacrificed at 60 d.p.i. were pre-stained with PNAd antibody, developed with alkaline phosphatase (pink), digested with PK, probed with POM1, and developed with BCIP/NBT (black). Low resolution images of histoblots revealed some prion-infected HEVs, some non-infected HEVs, and some PrP^Sc^ deposits that were not HEV-associated.
(TIF)

Figure S8 **No co-localization between dendritic cells and PrP in prion-infected** ***TNFR1^−/−^*** **-Ig lymph nodes.** Frozen sections from prion-infected TNFR1^−/−^-Ig lymph nodes were analyzed by immunofluorescence with POM1 (PrP; green; A), peripheral node addressin (PNAd; blue; B), and a dendritic cell marker (CD11c; red; C). No co-localization between PrP and CD11c was identified (D; overlay). Size bar = 100 µm.
(TIF)
