# Supplementary figures and images for: Lymphotoxin, but Not TNF, Is Required for Prion Invasion of Lymph Nodes
Source: PLoS Pathog. 2012 Aug 9;8(8):e1002867. doi: 10.1371/journal.ppat.1002867 (PMC3415451; doi:10.1371/journal.ppat.1002867)

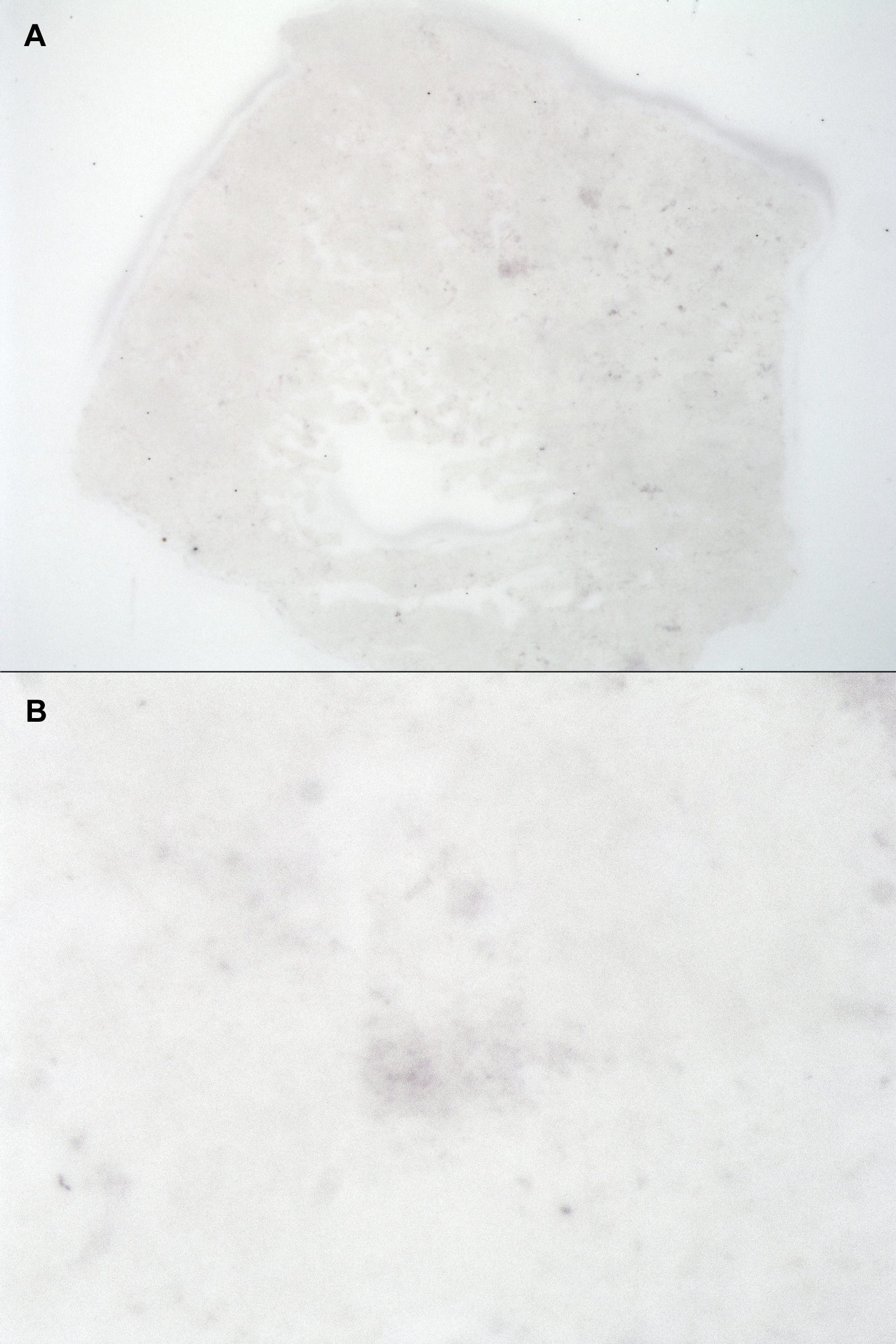

Supplement: Figure S1 — Low basal PrP immunoreactivity in histoblots from spleens of uninfected wild-type mice. Histoblots were performed on frozen sections from uninfected C57BL/6 (WT) spleens to determine the background level of PrPSc staining in tissue devoid of PrPSc. (A) Whole organ. (B) High resolution image. (TIF) [file ppat.1002867.s001.tif]

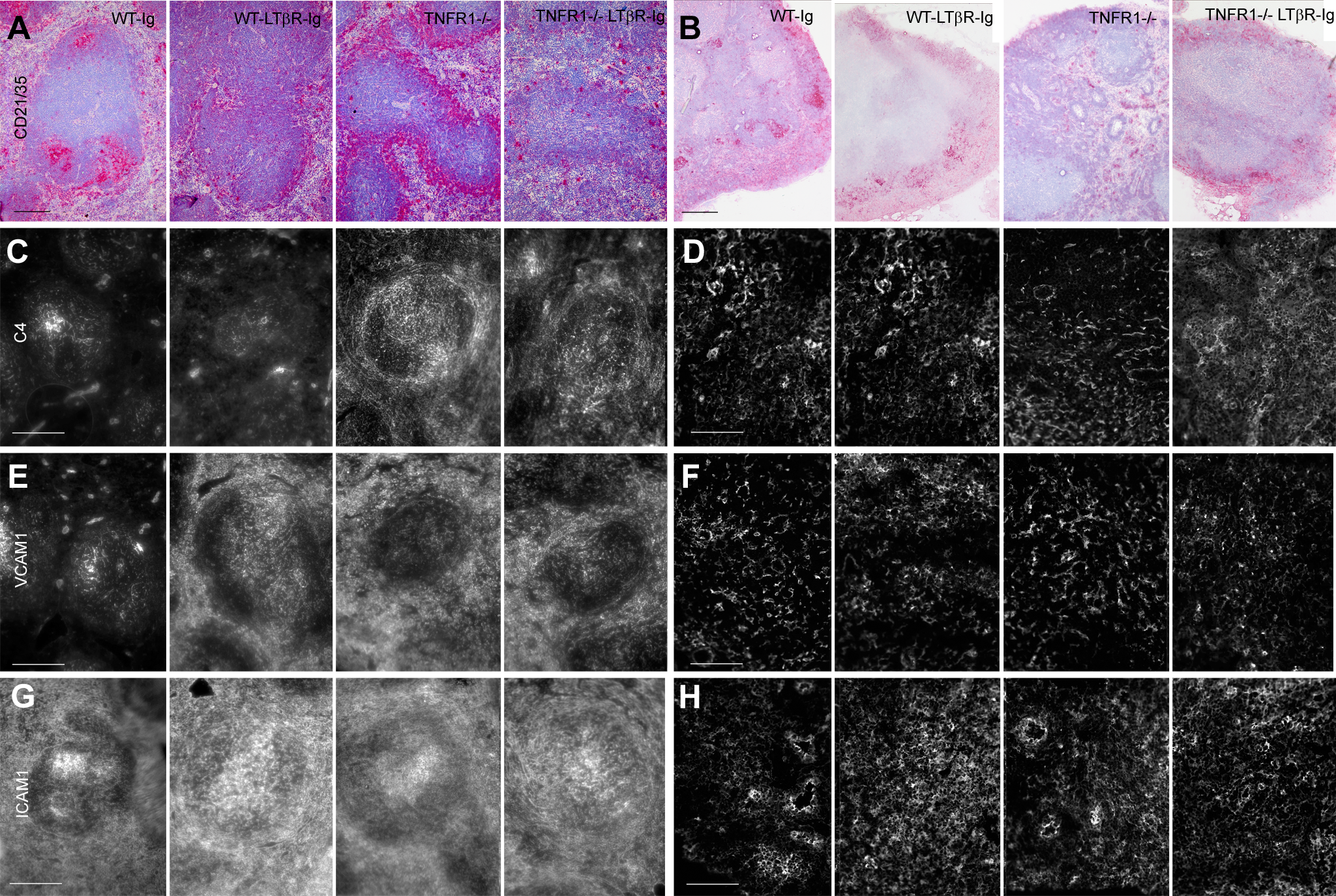

Supplement: Figure S2 — Common stromal markers in lymphoid organs do not correlate with prion deposition. Frozen sections from spleens (A, C, E & G) and mesenteric lymph nodes (B, D, F & H) of C57BL/6 (WT) Ig-treated, C57BL/6 (WT) LTβR-Ig-treated, TNFR1−/− Ig-treated, or TNFR1−/− LTβR-Ig-treated mice were analyzed by immunohistochemistry and developed with alkaline phosphatase (A & B) or immunofluorescence (C–H) for CD21/35 (A & B), complement factor C4 (C & D), vascular cell adhesion molecule 1 (VCAM1; E & F), and intercellular adhesion molecule 1 (ICAM1; G & H). Size bars in A & C = 200 µm; C–H = 100 µm. (TIF) [file ppat.1002867.s002.tif]

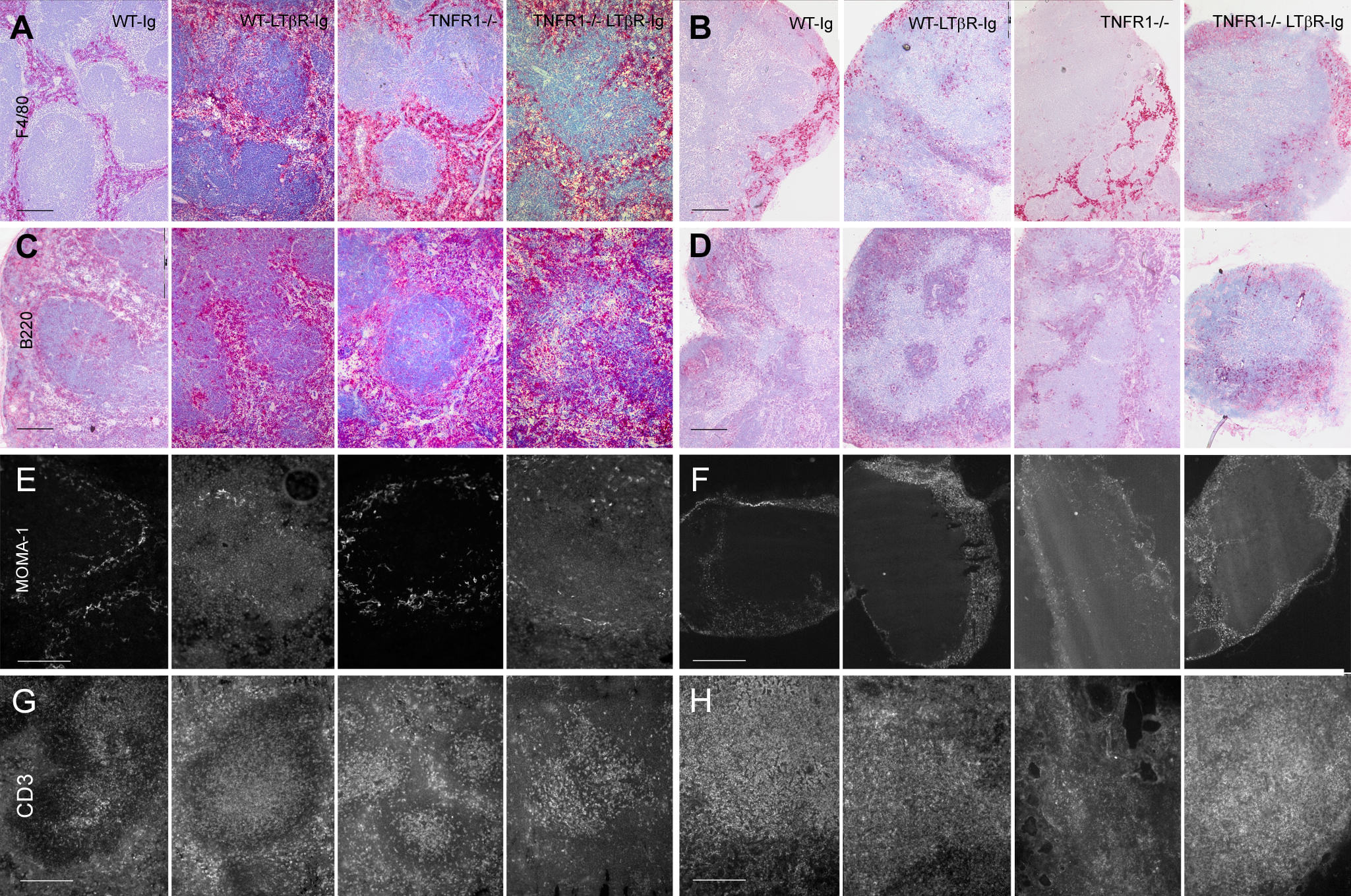

Supplement: Figure S3 — Common hematopoietic markers in lymphoid organs do not correlate with prion deposition. Frozen sections from spleens (A, C, E & G) and mesenteric lymph nodes (B, D, F & H) from C57BL/6 (WT) Ig-treated, C57BL/6 (WT) LTβR-Ig-treated, TNFR1−/− Ig-treated, or TNFR1−/− LTβR-Ig-treated mice were analyzed by immunohistochemistry and developed with alkaline phosphatase (A–D) or immunofluorescence (E–H) for macrophages (F4/80; A & B), B-cells (B-cells; C & D), metallophilic macrophages (MOMA-1; E & F), and T-cells (CD3; G & H). Size bars in A & C = 100 µm; B & D = 200 µm; E–H = 100 µm. (TIF) [file ppat.1002867.s003.tif]

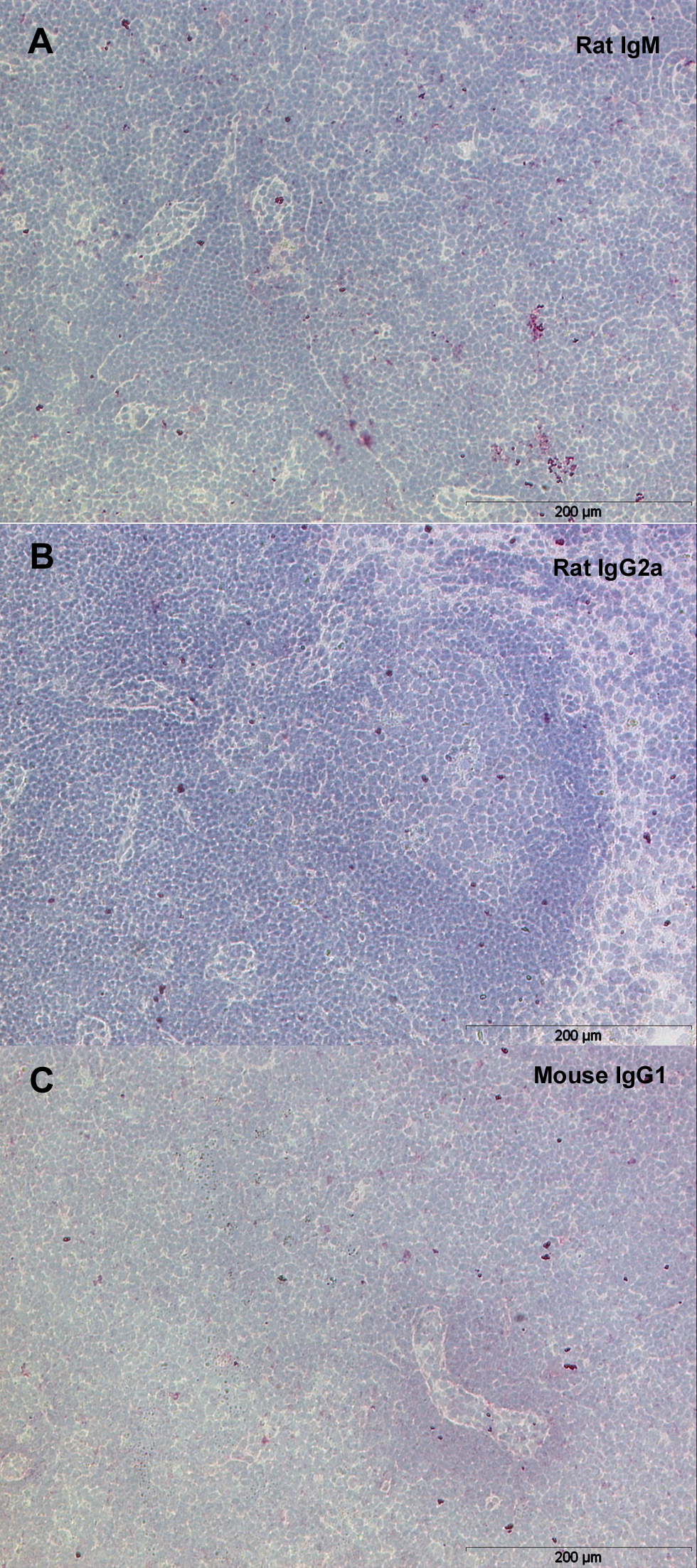

Supplement: Figure S4 — Neither rat nor mouse isotype controls immunoreact with mesenteric lymph node vessels or follicles. Frozen sections from mesenteric lymph nodes of C57BL/6 (WT) mice were immunostained with rat IgM (A), rat IgG2a (B), or mouse IgG1 (C) isotype controls and developed with alkaline phosphatase. No specific staining of vessels or follicles was observed. Size bars = 200 µm. (TIF) [file ppat.1002867.s004.tif]

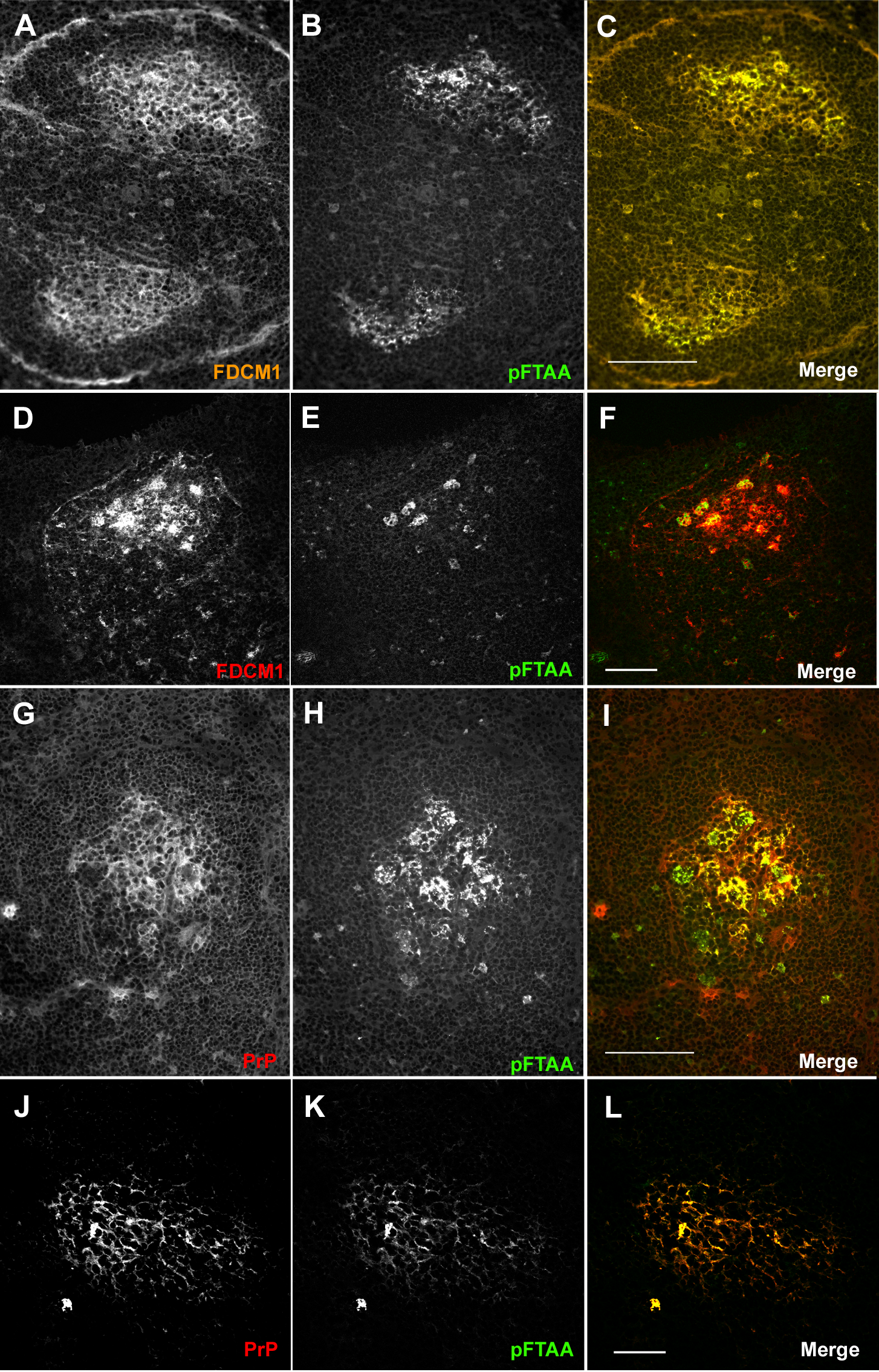

Supplement: Figure S5 — Pentameric formic thiophene acetic acid detects prion-infected FDC networks in spleens. Frozen sections from spleens of prion-infected C57BL/6 mice were analyzed by standard (A–C; G–I) or confocal (D–F; J–L) immunofluorescence with follicular dendritic cell marker 1 (FDCM1; orange; A & red; D) or prion protein antibody POM1 (PrP; red; G & J) and pentameric formic thiophene acetic acid (p-FTAA; green; B, E, H & K). p-FTAA co-localizes with PrP-positive (I & L; overlay) FDC networks (C & F; overlay) of prion-infected mice. Size bars in C & I = 100 µm; size bar in F = 60 µM; size bar in L = 40 µm. (TIF) [file ppat.1002867.s005.tif]

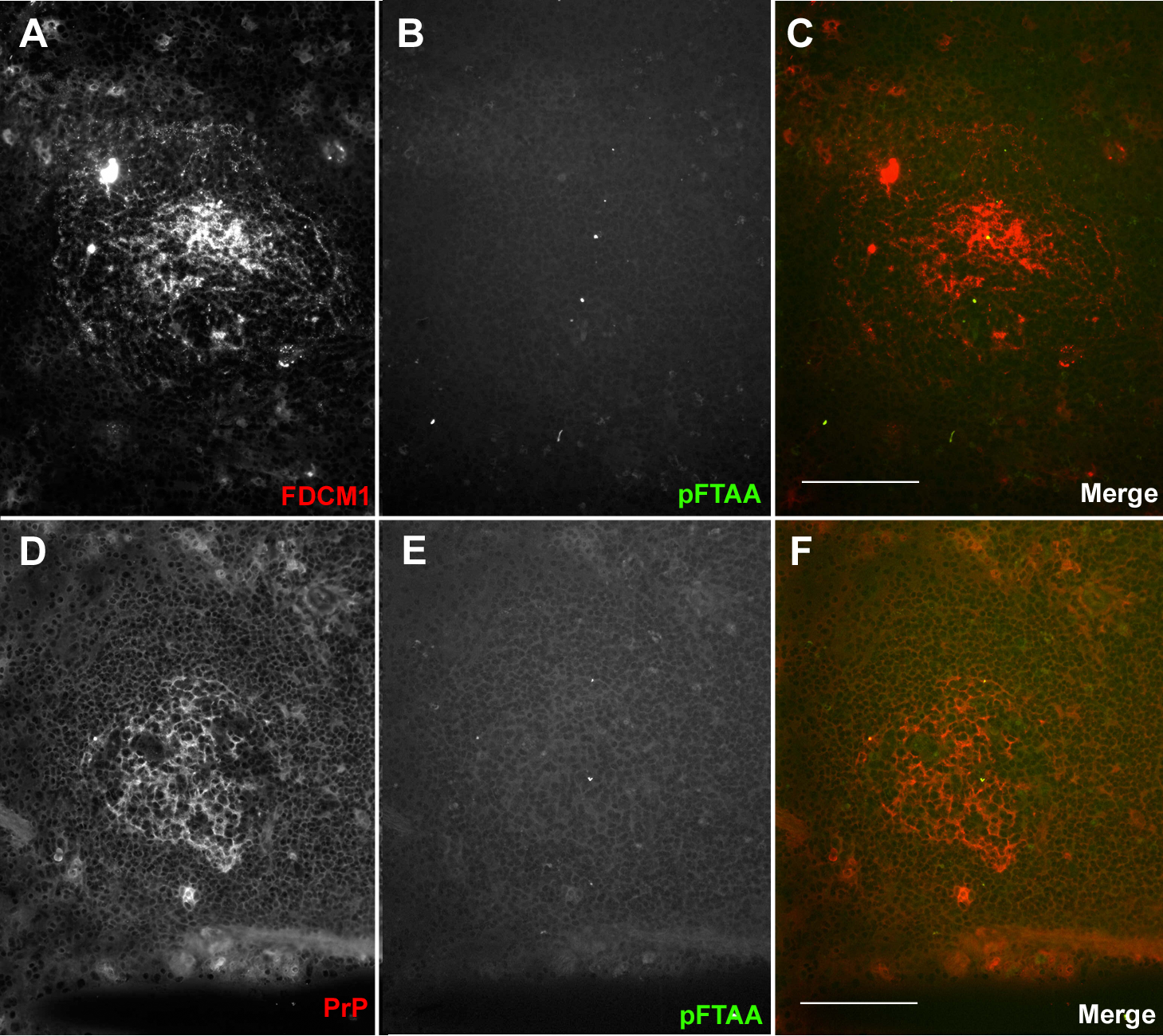

Supplement: Figure S6 — Pentameric formic thiophene acetic acid does not detect PrP-positive FDC networks in uninfected spleens. Frozen sections from spleens of uninfected C57BL/6 mice were analyzed by standard immunofluorescence with follicular dendritic cell marker 1 (FDCM1; red; A) or prion protein antibody POM1 (PrP; red; D) and pentameric formic thiophene acetic acid (p-FTAA; green; B & E). No p-FTAA staining was detected in PrPC-positive (C; overlay) FDC networks (F; overlay) of uninfected mice. Size bars = 100 µm. (TIF) [file ppat.1002867.s006.tif]

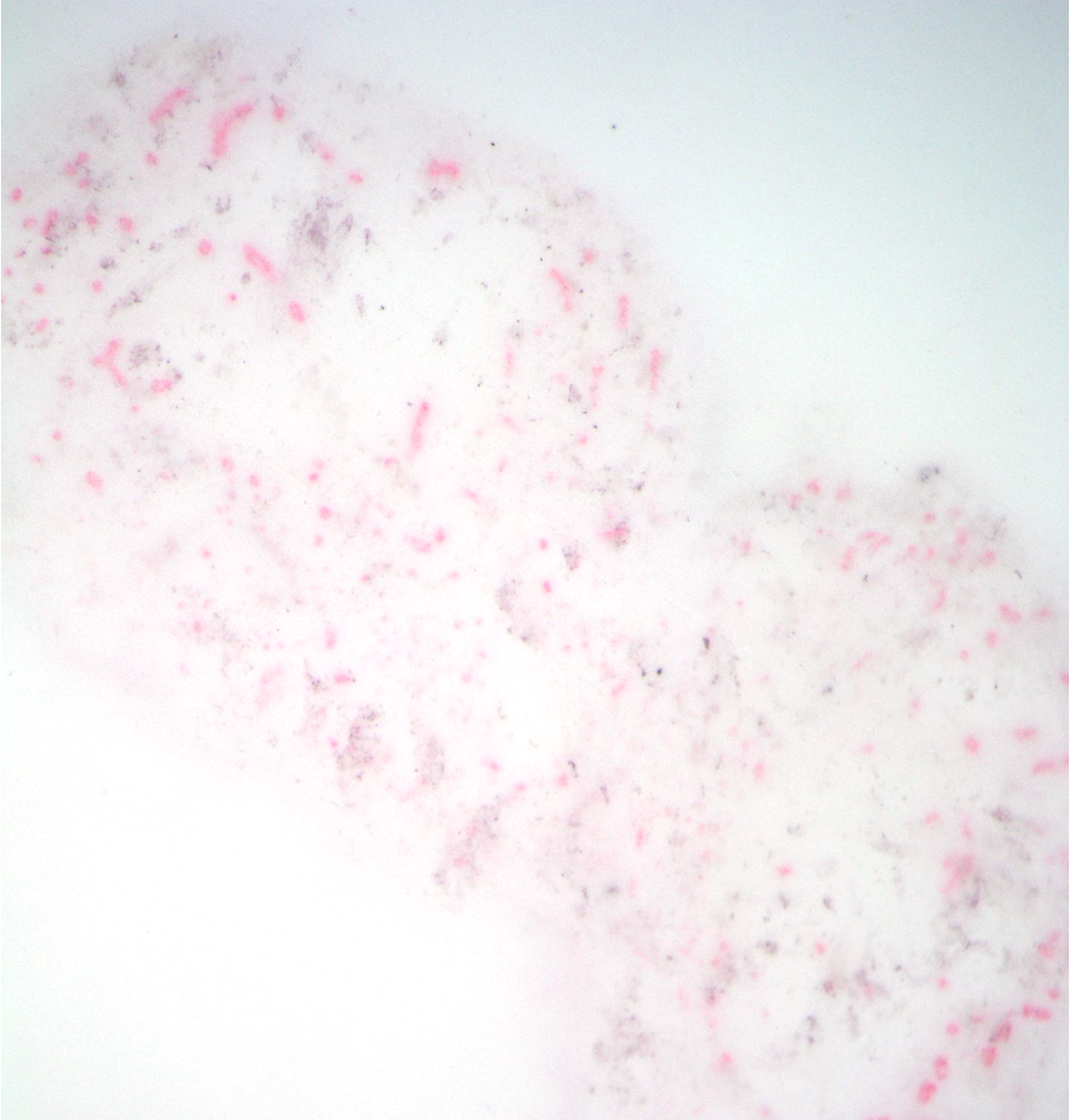

Supplement: Figure S7 — Tissue-wide analysis of PNAd pre-stained histoblots from prion-infected TNFR1−/− -Ig mLNs show that a proportion of PrPSc deposits co-localize to HEVs. Histoblots of mesenteric lymph nodes from TNFR1−/− mice inoculated i.p. with 6 log LD50 RML6, treated weekly with control Ig, and sacrificed at 60 d.p.i. were pre-stained with PNAd antibody, developed with alkaline phosphatase (pink), digested with PK, probed with POM1, and developed with BCIP/NBT (black). Low resolution images of histoblots revealed some prion-infected HEVs, some non-infected HEVs, and some PrPSc deposits that were not HEV-associated. (TIF) [file ppat.1002867.s007.tif]

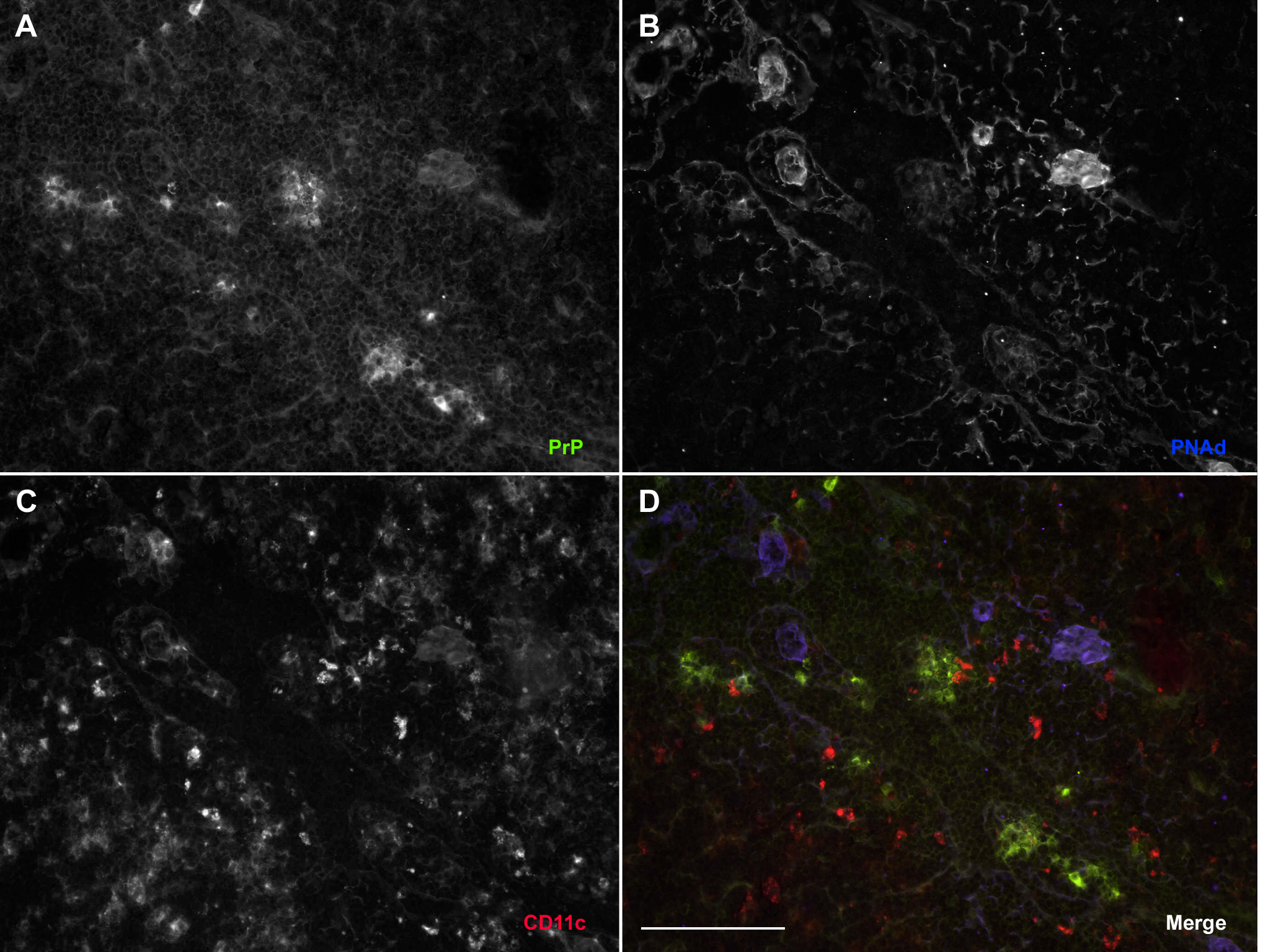

Supplement: Figure S8 — No co-localization between dendritic cells and PrP in prion-infected TNFR1−/− -Ig lymph nodes. Frozen sections from prion-infected TNFR1−/−-Ig lymph nodes were analyzed by immunofluorescence with POM1 (PrP; green; A), peripheral node addressin (PNAd; blue; B), and a dendritic cell marker (CD11c; red; C). No co-localization between PrP and CD11c was identified (D; overlay). Size bar = 100 µm. (TIF) [file ppat.1002867.s008.tif]
